# Development of International Terminology and Definitions for Texture-Modified Foods and Thickened Fluids Used in Dysphagia Management: The IDDSI Framework

**DOI:** 10.1007/s00455-016-9758-y

**Published:** 2016-12-02

**Authors:** Julie A. Y. Cichero, Peter Lam, Catriona M. Steele, Ben Hanson, Jianshe Chen, Roberto O. Dantas, Janice Duivestein, Jun Kayashita, Caroline Lecko, Joseph Murray, Mershen Pillay, Luis Riquelme, Soenke Stanschus

**Affiliations:** 1 International Dysphagia Diet Standardisation Initiative (IDDSI) Working Committee, Brisbane, QLD Australia; 2 School of Pharmacy, Pharmacy Australia Centre of Excellence (PACE), The University of Queensland, 20 Cornwall St, Brisbane, QLD 4102 Australia; 3 Faculty of Land and Food Systems, University of British Columbia, Vancouver, BC Canada; 4 Peter Lam Consulting, Vancouver, BC Canada; 5 Toronto Rehabilitation Institute, University Health Network, Toronto, ON Canada; 6 Rehabilitation Sciences Institute, Faculty of Medicine, University of Toronto, Toronto, ON Canada; 7 Department of Mechanical Engineering, University College London, London, UK; 8 Zhejiang Gongshang University, Hangzhou, China; 9 Medical School of Ribeirão Preto, University of São Paulo, Ribeirão Preto, Brazil; 10 Access Community Therapists, Vancouver, BC Canada; 11 Faculty of Medicine, University of British Columbia, Vancouver, BC Canada; 12 Department of Health Sciences, Prefectural University of Hiroshima, Hiroshima, Japan; 13 National Health Service Improvement, London, UK; 14 Ann Arbor Veterans Affairs, Ann Arbor, MI USA; 15 Speech Pathology, School of Health Sciences, University of KwaZulu-Natal, Westville Campus, Durban, South Africa; 16 Manchester Metropolitan University, Manchester, UK; 17 Department of Speech-Language Pathology, New York Medical College, Valhalla, NY USA; 18 Barrique Speech-Language Pathology at Center for Swallowing & Speech-Language Pathology, New York Methodist Hospital, Brooklyn, NY USA; 19 Swallowing and Speech Pathology, Hospital zum Heiligen Geist, Kempen, Germany

**Keywords:** Deglutition, Deglutition disorders, Swallowing, Dysphagia diet, Texture-modified food, Thickened fluid, Food and fluid standards

## Abstract

Dysphagia is estimated to affect ~8% of the world’s population (~590 million people). Texture-modified foods and thickened drinks are commonly used to reduce the risks of choking and aspiration. The International Dysphagia Diet Standardisation Initiative (IDDSI) was founded with the goal of developing globally standardized terminology and definitions for texture-modified foods and liquids applicable to individuals with dysphagia of all ages, in all care settings, and all cultures. A multi-professional volunteer committee developed a dysphagia diet framework through systematic review and stakeholder consultation. First, a survey of existing national terminologies and current practice was conducted, receiving 2050 responses from 33 countries. Respondents included individuals with dysphagia; their caregivers; organizations supporting individuals with dysphagia; healthcare professionals; food service providers; researchers; and industry. The results revealed common use of 3–4 levels of food texture (54 different names) and ≥3 levels of liquid thickness (27 different names). Substantial support was expressed for international standardization. Next, a systematic review regarding the impact of food texture and liquid consistency on swallowing was completed. A meeting was then convened to review data from previous phases, and develop a draft framework. A further international stakeholder survey sought feedback to guide framework refinement; 3190 responses were received from 57 countries. The IDDSI Framework (released in November, 2015) involves a continuum of 8 levels (0–7) identified by numbers, text labels, color codes, definitions, and measurement methods. The IDDSI Framework is recommended for implementation throughout the world.

**Electronic supplementary material:**

The online version of this article (doi:10.1007/s00455-016-9758-y) contains supplementary material, which is available to authorized users.

## Introduction

Standardized terminology exists to reduce misunderstanding and ambiguity and to improve communication efficiency [1]. The field of dysphagia has benefited from standardized scales in outcome measurement that allow clinicians to reliably document change in status during management. Examples of dysphagia-specific standardized scales include the Penetration–Aspiration Scale [2]; the Swal-QOL and Swal-CARE [3]; the Dysphagia Outcome Severity Scale [4]; and the Functional Oral Intake Scale [5]. However, despite the fact that texture modification is one of the most common intervention approaches for dysphagia [6], the descriptions of thickened drinks and texture-modified foods vary throughout the world, including within countries, and even across hospitals located with close geographic proximity [7]. We hypothesize that a standardized framework for dysphagia diets could offer benefits including but not limited to improved patient safety; improved communication within and between health professionals, healthcare providers and patients; increased visibility of professional interventions; and greater opportunities to collect and evaluate treatment outcomes [7–11]). Of these, the two most compelling reasons to pursue standardization of dysphagia diets are to promote patient safety and to facilitate evolution of the field to deliver better treatment outcomes.

Much like dose-driven medication prescriptions for different severities of medical conditions, individuals with dysphagia are assessed and prescribed graded food textures and drink thicknesses that are commensurate with their physical and cognitive abilities. Also similar to medication adverse events, inconsistencies and errors in labeling of texture-modified foods have unfortunately resulted in deaths attributed to the delivery of inappropriate food textures to patients with dysphagia [7, 12–14]. In recent years, a number of countries have worked hard to create standards for texture-modified foods and thickened drinks with the goal of improving patient safety and care [7, 15–20]. However, with an increase in mobility of the global community and access to information via the internet, the plethora of dysphagia diet terminology, labels, numbers, and levels of food texture and thickened drinks has only led to greater opportunities for confusion. Furthermore, a proliferation of companies producing thickeners and ready-to-use products means that patients and their caregivers cannot assume similarity in thickness across brands. This scenario is in contrast to expectations of bioequivalence in medicine between name brand medications and generic versions, which must have the same active ingredient, strength, dosage form, and route of administration as the brand name product [21]. There is no such regulation of dysphagia products to ensure ‘like-for-like’ in terms of commercially prepared fluid thickness levels or food texture modification. To be fair, manufacturers cannot be held accountable for producing products that conform to standards until such standards have been established, and this requires stakeholder agreement, clarity regarding labels, detailed definitions, and testing methods to demonstrate conformance to desired properties.

A lack of standardized nomenclature regarding food texture and drink thickness is a major barrier to research in the dysphagia field. Without clear definitions, we cannot presume that the outcomes of research conducted on the efficacy of prescribing ‘nectar-thick’ drinks for patients with Parkinson’s disease in one country, for example, can be generalized around the world. Use of a term such as ‘nectar-thick’ in research conducted in the USA (e.g., [22, 23]) may not translate to products or liquid consistencies used in other countries, such as the United Kingdom, Japan, or Australia, regardless of the fact that each has a set of National Descriptors. Without agreement on a single standardized terminology, clinical research and development of therapies is impeded.

The International Dysphagia Diet Standardisation Initiative Inc. (IDDSI) was founded in 2012 by a multi-professional international group of volunteers. IDDSI is an independent, not-for profit entity (Incorporation Number IA40577). The ultimate objective of the initiative is to pursue a patient-safety-oriented innovation in practice, based on consideration of research evidence, current practice, and stakeholder feedback. There was no plan for the initiative to address the nutritional adequacy or the patient acceptability of texture-modified foods or thickened fluids.

The aims of the initiative that are discussed in this manuscript were to

1. determine the number of food texture and drink thickness levels for international standardized use (adult and pediatric);
2. develop culturally sensitive standard names/identifiers for each food and drink level;
3. develop detailed definitions for each level of food texture and drink thickness;
4. develop user-friendly, inexpensive, easily accessible measurement methods for determining classification of food textures and thickened drinks;
5. seek input and consensus from international stakeholders; and
6. publish and widely communicate the international standards.

The process used to develop the framework follows the key elements of evidence-based practice guideline development including those recommended by the National Health and Medical Research Council of Australia (NHMRC), the National Institute of Health and Clinical Excellence in the UK (NICE), the New Zealand Guidelines Group (NZGG), the Scottish Intercollegiate Guideline Network (SIGN), the Council of Europe, and the World Health Organization (WHO) [24]. These key elements include: establishment of a multidisciplinary guideline development group; involvement of consumers; clear identification of clinical issues; systematic review and appraisal of quality literature; a process for drafting the recommendation of the multidisciplinary group; and consultation with others beyond the multidisciplinary framework development group [24, 25].

## Methods

### Strategy and Incorporation

An inaugural multi-professional expert panel meeting was held in Toronto, Canada (2012) to discuss international standardization of terminology and definitions for texture-modified foods and drinks. A snowball sampling methodology was used to populate the expert panel following initial recruitment of two members with experience in the development of national terminologies [7, 19] and one who had commenced but not completed national terminology development in Canada (PL). Remaining members were invited to join the panel based on their previous work with national guideline development [16], their representation of key stakeholder groups, and their ability to contribute international perspectives. In 2013, IDDSI was incorporated as an independent, not-for-profit association operating under the regulatory guidelines of its registration in Australia. Two people volunteered to be co-chairs, with ten others agreeing to serve as members of the IDDSI Foundation Committee. All positions were voluntary. The committee, representing ten countries, was composed of experts from the fields of Nutrition and Dietetics, Food Service and Catering, Speech Pathology, Occupational Therapy, Physiotherapy, Gastroenterology, Nursing, Mechanical Engineering, and Food Science. The group counted among its members published scientists, journal editors, representatives from international organizations such as the Patient Safety - Nursing Directorate, National Health Service (NHS) England, and internationally recognized dysphagia clinicians and researchers. The committee met by teleconference on a monthly basis, with two in-person meetings over the project period (2013–2015). Sponsors were approached for financial support to cover costs associated with administration, research, and data analysis (e.g., research assistant support for the systematic review). At no time have sponsors been involved with the design or development of the IDDSI framework; rather, IDDSI sponsors have been briefed about IDDSI progress using time-zone sensitive teleconferences at key milestones over the course of the IDDSI project. Professional associations and organizations were also contacted to alert them to the IDDSI project and invite their participation and support.

**Fig. 1:**
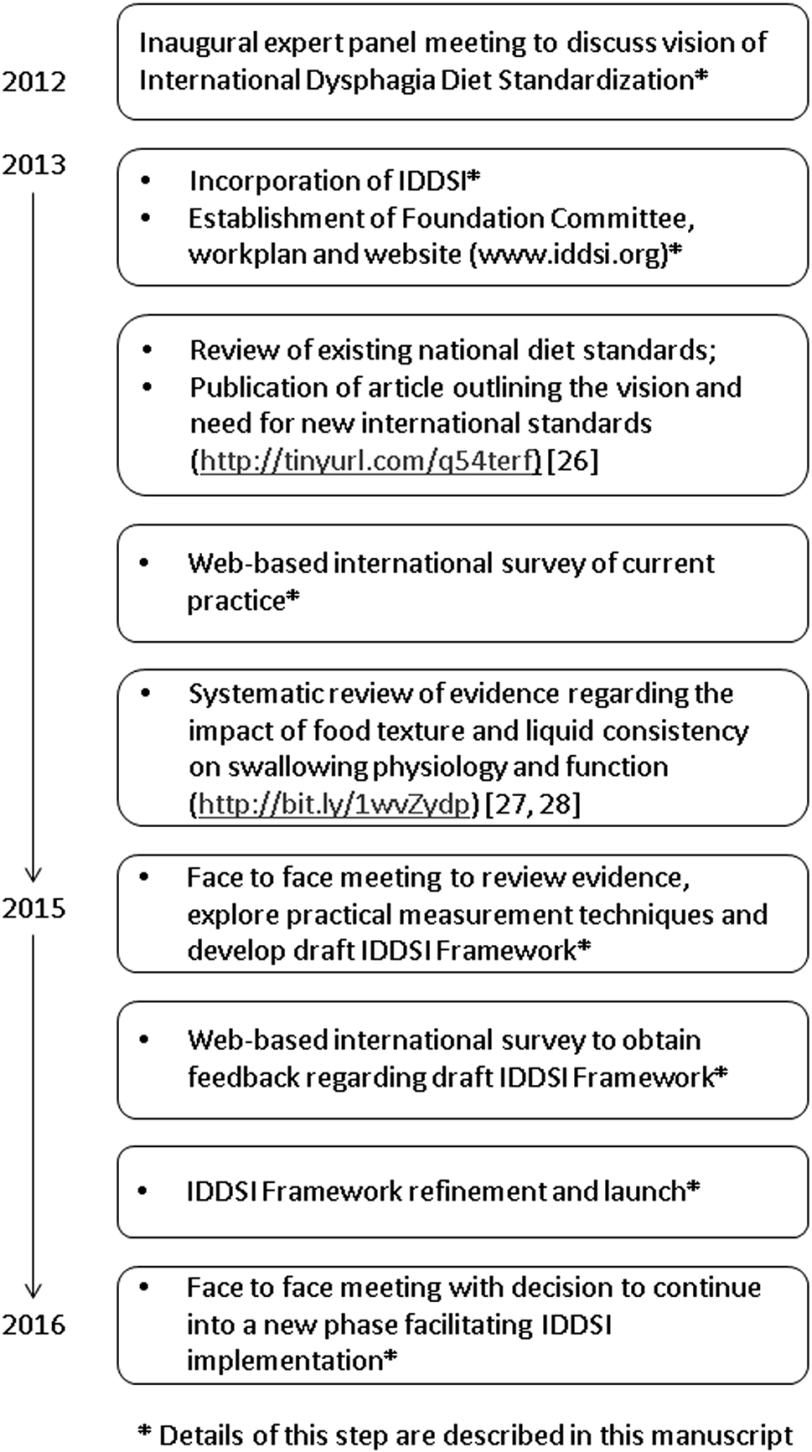
Timeline of the international dysphagia diet standardisation initiative

A dedicated website was developed to provide an internationally accessible repository for information and a way for interested individuals or groups to contact IDDSI (www.iddsi.org). The IDDSI project plan, committee member profiles, lists of supporting organizations, and sponsors can be found on the website. A multi-phased work plan was approved by the committee with the goal of bringing forth a framework between 2013 and 2015. Each of these phases is summarized in Fig. 1.

### Ethical Considerations

The IDDSI committee considered ethical issues associated with the collection of survey data in different phases of the project. It was agreed that participation in IDDSI surveys involved minimal risk and was entirely voluntary. The committee agreed that the purpose of each survey and the overall project would be communicated at the time of invitations to participate. The introductory text of IDDSI surveys stated clearly that information gathered from individuals or organizations would remain non-identifying in all reports arising from the project. Participants were also free to withhold responses to survey questions at any stage without penalty. Several key stakeholder groups were identified and attempts were made to disseminate invitations regarding survey opportunities to all of these groups. To avoid commercial conflicts of interest, it was agreed that industry sponsors would not be involved with any aspects of IDDSI sub-project design, conduct, writing, or interpretation of results.

### Review of Existing National Terminologies

A review of existing national terminologies was conducted and published in 2013. Further details can be found in the open access journal publication [26].

### Survey 1 (International Current Practice)

In 2013, a set of five stakeholder-specific surveys was developed to gather information regarding the current use of standardized dysphagia diet terminology or other terms used, any testing done prior to serving to ensure correct consistency/thickness and appropriate texture, use of schemes to differentiate levels (e.g., colors, shapes), and comments or recommendations for the development of an international standardized framework. Each survey was tailored to one of five stakeholder groups: (a) individuals with dysphagia, their caregivers, or organizations providing support to people with dysphagia; (b) healthcare professionals and food service professionals; (c) dysphagia research scholars; d) industry representatives from companies manufacturing texture-modified foods; and (e) industry representatives from companies manufacturing thickeners or thickened drinks for people with dysphagia. English language style and complexity of the surveys was adjusted to be appropriate for each group. There were commonalities in some survey questions, while other elements were specific to stakeholder experiences (see Table 1).

**Table 1:** Questions included in the current international practice survey by stakeholder group

Question	Response options	Patients and their carers or support organizations	Healthcare professionals and food service staff	Dysphagia research scholars	Industry representatives
What country are you located in?	Free text	X	X	X	X
How would you describe yourself?	An adult with dysphagia	X			
	I care for an adult with dysphagia	X			
	I care for a child with dysphagia	X			
	I am a member of an organization that specifically supports people with dysphagia	X			
	I am a member of an organization that supports children with dysphagia	X			
	Healthcare professional (specify)		X		
	Food service or catering professional		X		
	Representative of company manufacturing thickeners, thickened liquids, or barium				X
	Representative of company manufacturing texture-modified foods				X
What is your work setting?	Hospital/acute care		X	X	
	Rehab hospital		X		
	Long-term care/Home for the aged		X		
	Community care		X		
	Outpatient clinic		X		
	University			X	
	Government or private laboratory			X	
Who are your patients/clients?	Adults		X		
	Children		X		
	Mixed age group		X		
What type(s) of dysphagia research do you conduct?	Clinical research into oropharyngeal dysphagia			X	
	Clinical research into esophageal dysphagia			X	
	Fundamental swallowing research			X	
	Product development			X	
	Other (specify)				
Do you/the person you care for/your patients use	Texture-modified foods	X	X		
	Commercially prepared texture-modified foods	X	X		
	Thickened liquids	X	X		
	Commercially prepared thickened liquids	X	X		
	A combination of the above	X	X		
Do you use standard terminology and guidelines for texture-modified foods and liquid thicknesses?	Yes/No/I don’t know with comments option	X	X		
Do you produce thickened liquids or products that will thicken liquids, for people with swallowing difficulties or those who are nutritionally compromised?	Ready-to-use drinks				X
	Powder thickeners				X
	Liquid thickeners				X
	Other (specify)				X
Do you produce thickened liquids, thickening agents, or barium products specifically for people with dysphagia?	Yes/No with comments option				X
Do you produce foods for people with chewing and swallowing difficulties or those who are nutritionally compromised?	Ready-to-eat frozen meals				X
	Ready-to-eat packaged foods (tins, pouches)				X
	Foods that need reconstituting				X
	Other (specify)				X
Do you produce texture-modified foods specifically for people with dysphagia?	Yes/No with comments option				X
Which terms do you use for texture-modified foods and liquid thicknesses	Terms for texture-modified foods (from least to most modified, separated by a comma)	X	X	X	X
	Terms for thickened liquids (from least to most modified, separated by a comma)	X	X	X	X
Where did you source the terms you use for thickened liquids and texture-modified foods?	National standards			X	X
	From the literature			X	X
	Self-developed			X	X
	Hospital or work facility			X	X
	Other (specify)			X	X
Do you test the consistency of foods or liquids before eating or serving?	Yes/No with comments option	X	X		
How do you determine whether liquids are of the correct thickness?	Free text			X	
How do you determine if foods are of the correct texture?	Free text			X	
Do you formally test thickened liquids or texture-modified foods as part of your research?	Liquids: always/usually/rarely/never with comments option			X	
	Food: always/usually/rarely/never with comments option			X	
	Barium: always/usually/rarely/never with comments option			X	
For thickened liquids (including barium), do you record information about:	Viscosity: always/usually/rarely/never with comments option			X	X
	Density: always/usually/rarely/never with comments option			X	X
	Yield stress: always/usually/rarely/never with comments option			X	X
If you formally evaluate thickened liquids or texture-modified foods, what measurement device(s) do you use? (For industry respondents, target measurement details were requested for each method)	Visual inspection				X
	Line spread test			X	X
	Bostwick consistometer			X	X
	Brookfield viscometer			X	X
	Cone and plate or parallel plate rheometer			X	X
	Other rheometer			X	X
	Food texture analyzer			X	X
	Sieve				X
	Image analysis				X
	Other (specify)			X	X
For texture-modified foods, do you record information about	Particle size: always/usually/rarely/never with comments option			X	X
	Cohesiveness: always/usually/rarely/never with comments option			X	X
	Adhesiveness: always/usually/rarely/never with comments option			X	X
	Firmness: always/usually/rarely/never with comments option			X	X
	Springiness: always/usually/rarely/never with comments option			X	X
	Brittleness or fracturability: always/usually/rarely/never with comments option			X	X
	Hardness: always/usually/rarely/never with comments option			X	X
	Yield stress: always/usually/rarely/never with comments option			X	X
	Other (specify)			X	X
Do you use a scheme (colors, numbers, shapes, etc.) to differentiate/communicate the different food textures and liquid thicknesses?	Yes/No with comments option	X	X		X
What problems, if any, are there with the terminology or definitions you currently use for thickened liquids or texture-modified foods?				X	X
Where do you distribute your products?	Locally/regionally/nationally/internationally with product type identified				X
Are there any other comments or recommendations you would like to make for the International Dysphagia Diet Standardisation Initiative?	Free text	X	X	X	X
Additional optional information	Name and address for future contact	X	X	X	X

Surveys comprised forced choice and free-text response formats. An information sheet about the survey and invitation to participate were translated by native speaker volunteers into 10 languages other than English. Surveys were launched via the IDDSI website to individuals who had signed up to receive information about the initiative. In addition, 45 national healthcare professional associations and three dysphagia-specific associations were emailed information about the survey and asked to forward notices to their membership with embedded web links to facilitate ease of survey access. Invitations to complete the survey were also announced at international conferences. Survey responses were collected from October 2013 to November 2014 using Surveymonkey™. Upon closure of the surveys, the response data were transferred to an independent research group for analysis (Australian Survey Research Group) in order that the IDDSI committee did not have any opportunity to inadvertently bias the results.

### Systematic Review of the Literature

A systematic review of the literature regarding the influence of food texture and liquid consistency on swallowing physiology was conducted in 2014, with the results published in 2015. The key findings from the systematic review showed that there is evidence that thicker liquids not only reduce the risk of penetration–aspiration, but also increase the risk of post-swallow residue in the pharynx. Further, the existing literature is insufficient to support the delineation of specific viscosity boundaries or other quantifiable material properties related to clinical outcomes. With regards to food texture used in dysphagia management, the systematic review determined that the best available evidence for selecting optimal food consistency comes from careful exploration of tolerance for different foods as part of a comprehensive swallowing assessment. The systematic review also demonstrated evidence that solid food and thick consistencies require greater effort in oral processing and swallowing. Note that terms related to choking, airway obstruction, or asphyxiation were not included in the search strategy for the systematic review. Further details can be found in the open access journal publication [27, 28].

### Draft Framework Development

With information gathered from (a) existing national dysphagia diet terminology from around the world [26]; (b) the current practice international stakeholder surveys; and (c) the systematic review [27, 28], the IDDSI committee gathered in Vancouver, Canada in January, 2015 for a 2 ½ day in-person expert panel meeting to develop a draft international framework. Committee members from Australia, Canada, Germany, Japan, and the United Kingdom were able to attend in person, covering areas of expertise in nutrition and dietetics, food service and catering, speech pathology, occupational therapy, physiotherapy, food science, mechanical engineering, research, and both adult and pediatric clinical dysphagia services. Input from absent committee members on key questions was obtained via e-mail and telephone both during the meeting and over the following months.

The objectives of the expert panel meeting were to determine

- the number of levels of texture-modified foods for inclusion in a new standardized international dysphagia diet framework;
- the number of levels for thin and thickened drinks for inclusion in a new standardized international dysphagia diet framework;
- English language labels for texture-modified foods at each level;
- English language labels for thickened drinks at each level;
- a numbering system;
- whether to use a color scheme;
- graphical representations to capture the framework;
- detailed definitions and descriptions of the texture or flow characteristics of food and drink items included at each level; and
- reproducible testing methods to enable end users to assign foods and drinks to the different levels.

A group nomination process was used to achieve decisions for objectives a) to g). After discussion of the available evidence (both from the scientific literature and collected through the current international practice survey), motions were put forward and committee members indicated their agreement or dissent through a blinded ballot process. Unanimous voting resulted in adoption of that particular motion. Less than unanimous voting resulted in further rounds of discussion and further blinded voting until unanimous consensus was reached. There were only two occasions where a second round of voting was required.

### Classification of levels and exploration of measurement methods

Based on consideration of the scientific and survey evidence, the IDDSI Committee achieved consensus that a new framework should include 5 levels of drink thickness (thin plus 4 levels of thickness) and 5 levels of food texture (regular plus 4 levels of modification). The next aim was to define and describe the specific texture/flow characteristics for each level.

#### Liquids

**Fig. 2:**
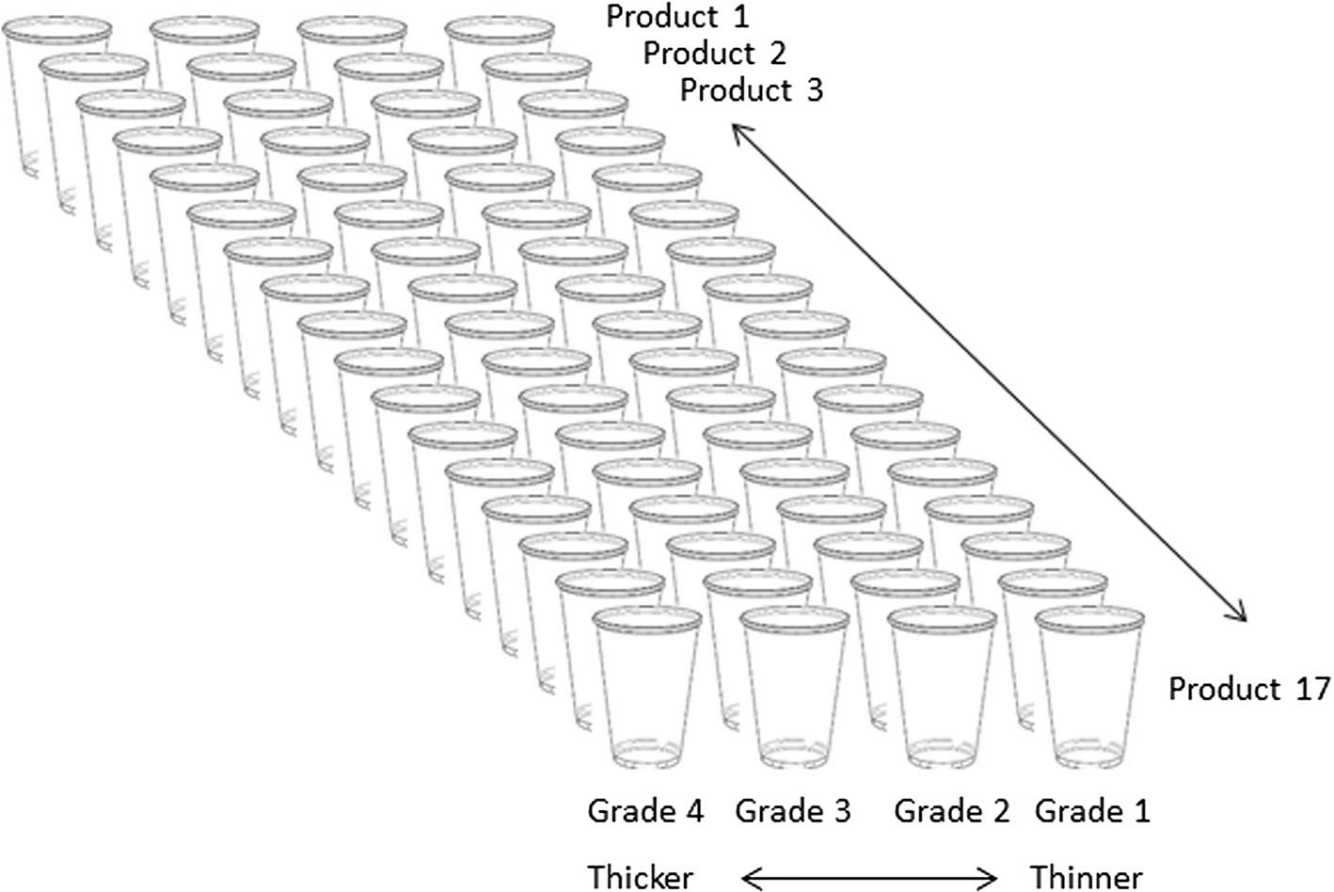
Set up of thickened liquids for comparison and evaluation

Thirteen powder, gel, or liquid-thickening agents and four brands of commercially pre-prepared thickened liquids (produced by manufacturers from Australia, Canada, Japan, the United Kingdom and the United States of America) were either donated or purchased prior to the meeting. Thickening agents included starch, gums, or combinations of starch and gums. Participants at the face-to-face meeting worked in pairs to prepare samples of graded thickness according to the manufacturer’s instructions. The various thickening agents were mixed with cranberry juice (Ocean Spray). Previous national guidelines have identified a need for dysphagia diet frameworks to include a level for thickened infant milk, which is thinner than the first level of thickness commonly used for adults, but will still flow through a nipple/teat [7]. Therefore, in cases where the manufacturer’s instructions on products typically used for adults specified only three gradations of thickening, an additional level between thin and the first level of thickening was prepared to produce a thickness akin to thickened infant milk. Half of the manufacturer recommended the amount of thickener for the first level of thickened drinks was used to prepare this new thickness level. Samples of human milk and infant formulas, including specialty anti-regurgitation, semi-elemental, and elemental formulas were also prepared and thickened with a view to ensuring that the framework would address needs across the pediatric-to-adult continuum. The resulting array of samples comprised four columns by 17 rows of liquid, such that the far left column was the thickest item and the far right column was the thinnest for that product according to the manufacturer’s instructions. This array, which is illustrated by the schematic diagram in Fig. 2, allowed comparison of consistency across the nominally similar items in each row. Participants then continued to work in pairs to evaluate, measure, and describe the flow characteristics of all items in the array.

As well as assessing the consistency of products in each level, participants concurrently assessed the efficacy and reliability of several available methods for subjective and objective measurement, including: visual inspection; stirring; pouring from a spoon or cup; oral sampling and tasting; the line spread test [29]; and gravity flow tests using drinking straws and syringes of various dimensions. Rheological data were not obtained at the time, however, some members of the committee had familiarity with existing rheological measures for some of the products present. Based on these extensive tests, clusters of similarly behaving liquids were created. Further testing and discussion enabled the committee to confirm the similarity of liquids in each of five clusters corresponding to five levels of drink thickness (including thin) and to develop descriptions of the flow characteristics of each level. The syringe-based flow test was confirmed to be the preferred testing method for quantifying liquid consistency and it was agreed that four members of the committee with food science expertise would do further testing of this method upon return to their cities of origin to confirm construct validity in comparison to laboratory rheology and to establish boundary points between levels for liquids with different flow characteristics. This subsequent verification testing led to finalization of the IDDSI Syringe Flow Test (see Results section, below). The syringe-based flow test also affords the ability to evaluate liquids that are not typically considered ‘drinks’ such as condiments (e.g., sauces), liquid foods (soups), and nutritional supplements or liquid medication. The text term ‘thickened liquids’ is intended to include all of these items in addition to thickened drinks.

#### Foods

In order to develop a system for categorizing food texture, labels and descriptors for five different levels were proposed through a group nomination process. A hotel chef (naïve to dysphagia and texture-modified food used for this population) was then asked to prepare foods from the hotel menu in consistencies matching the draft labels. These samples, together with samples of ready-to-use texture-modified foods donated by industry, were assessed by the committee using spoons and forks (dropping and pressure tests) and oral appraisal, providing the opportunity to consider mouthfeel and the behavior of the sample in the mouth.

Through debate to the point of group consensus, the committee developed definitions of thickened liquids and texture-modified foods, together with the physiologic rationale for each level in the draft framework. Descriptors and physiologic rationale for each level were based on the shared experience of experimenting with a very broad range of currently available dysphagia products, combined with each expert’s relevant experience, and drawing from descriptions in all available national standard documents. Proposed labels were assessed via readability scores (Flesch–Kincaid Reading Ease score [30]) to confirm ease and understanding of terms in English. In addition, translation to languages other than English was achieved with assistance from personal contacts and volunteers, so that provisional translations of the terms were developed over the next month in Afrikaans, Arabic, Dutch, Farsi, French, German, Greek, Hebrew, Italian, Japanese, Korean, Mandarin, Portuguese, Spanish, Swedish, Turkish, and Vietnamese. Preferred methods of objective testing for liquids and foods were discussed with plans to review and finalize these following laboratory assessments upon return to cities of origin and a planned second international stakeholder survey of the draft framework. These steps led to a consensus-based and evidence-informed draft framework for public consultation, described in the Results section below.

### Survey 2: Feedback on Draft IDDSI Framework

A second international stakeholder online survey was designed with assistance from the Australian Survey Research group (ASR) to gather feedback on the draft framework. ASR administered, analyzed, and reported on the survey. The survey was announced and disseminated in the same way as the preceding current practice survey.

Respondents were specifically asked to use Likert-scale responses with additional free-text comment boxes to

- provide demographic information (e.g., the stakeholder group they identified with; country they lived/worked in);
- provide feedback regarding the draft framework:
  - colors (ability to distinguish; ease of implementation; ease of reproducibility);
  - number of levels (too few/too many, about right; ease of implementation);
  - pyramid diagram (ease of understanding and implementation);
  - names or labels of each level (ease of understanding and implementation);
  - specific questions about the terms “slightly thick” liquids, “minced and moist” food, and the label “Level 7 minus”;
  - detailed definitions of each level (ease of understanding; usefulness; relevance); and
  - the Syringe Test (ease of understanding; likelihood of implementing the test).
- Comment about the overall framework; and
  - what works well with the proposed framework; likelihood of implementation; and factors that would assist or impede implementation.

The online survey was open from May 1 to 1 June 1, 2015. The results of the stakeholder survey informed the final framework. Robust committee discussion followed via email and teleconferences between July–November 2015. The final framework comprises: (a) a diagram of the framework, including labels and colors; (b) detailed definitions and testing methods for liquids; and (c) detailed definitions and testing methods for foods (see Appendix in supplementary material).

## Results

### Survey 1 (Current International Practice)

**Fig. 3:**
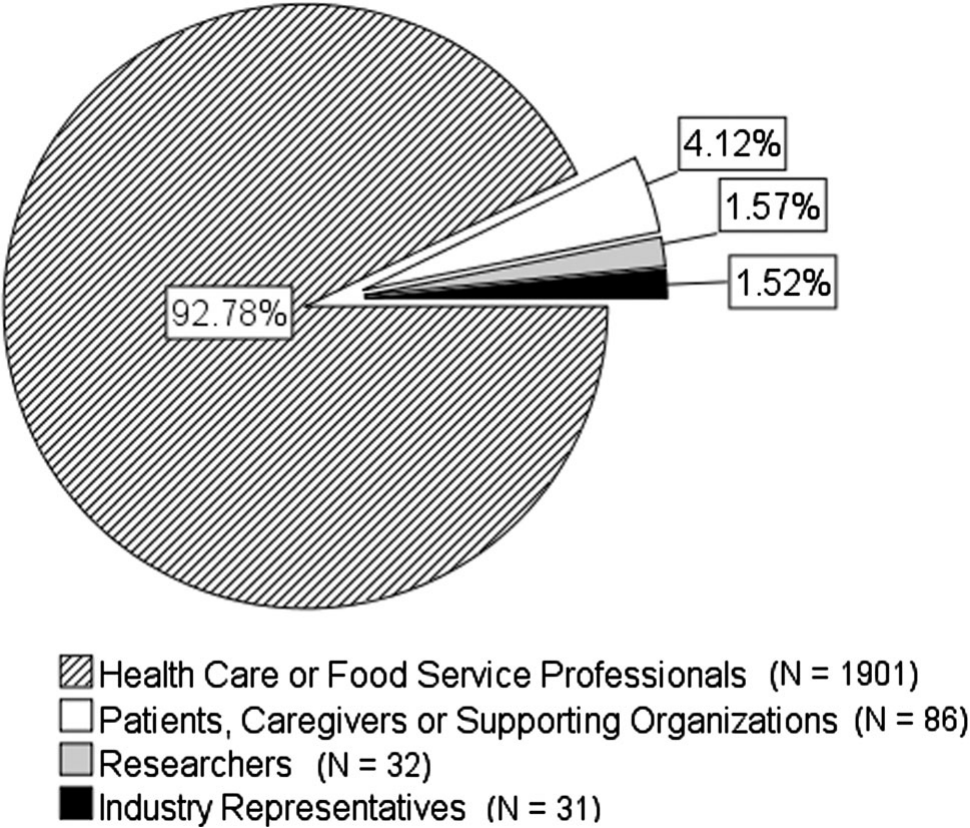
Distribution of survey respondents by stakeholder group

The current practice survey yielded responses from 2050 participants representing 33 countries. The majority of responses came from Canada, the USA, Australia, New Zealand, and the United Kingdom. Figure 3 illustrates the distribution of respondents by stakeholder group. Eighty percent of health professionals who responded saw adults with dysphagia, 8.2% saw children with dysphagia, and 16.5% saw a mixed caseload. Health professionals predominantly saw individuals in hospital settings (>60%) and approximately one quarter saw individuals in the community or aged care settings.

Healthcare professional respondents reported use of both site-prepared and commercial ready-to-use modified products. This was particularly true with respect to the preparation of texture-modified foods, for which fewer than 1% reported exclusive use either of commercial or on-site preparation methods. For drinks, exclusive use of commercially pre-thickened drinks was reported by 17% of respondents who had pediatric caseloads, and 30% of respondents whose caseloads included adults or a mix of adults and children. Exclusive in-house preparation of thickened drinks was more common for those working with pediatric caseloads (46%) compared to 30% for those working with adults or mixed caseloads.

Between 85 and 90% of health professionals reported using standardized terminology to describe thickened drinks and texture-modified food. However, considerable variation in terminology was observed from the responses obtained both within and between countries around the world. There were 27 different labels reported to be in use to refer to ≤ 5 levels of drink thickness. Most commonly, drink options were reported to include regular thin liquids plus three or more levels of thickened drinks (see Table 2). Of particular note, survey responses confirmed use with pediatric and palliative care clients of slightly thickened drinks that are thicker than water but thinner than the thickened drinks commonly used for adults [7, 11, 17, 31–34].

**Table 2:** Thickened drink names and number of levels by world region

Region	Names (least to most modified)
Africa	Normal/regular, nectar, syrup, pudding, thick
Australia + New Zealand	Thin, mildly thick/level 150, moderately thick/level 400, extremely thick/level 900
Asia	Thin, slightly thick, mildly thick, medium thick, extra thick
Canada	Thin, nectar, honey, pudding
Europe	Normal, syrup/slightly thick, nectar, honey, pudding
Ireland	Regular/normal, Gr 1, Gr 2, Gr 3, Gr 4
Middle East	Thin, mildly thick, moderately thick, other thick
South America	Liquid, slightly thick, nectar, honey, pudding
United Kingdom	Normal, stage 1, syrup, custard, pudding/stage 3
United States of America	Thin, nectar, honey, pudding

*Note* 27 different labels were identified internationally for ≥ 5 liquid thickness levels

For texture-modified foods, a total of 54 labels were reported to be in use to refer to ≤5 levels. Food options were reported to commonly include regular, non-modified foods plus four to five levels of texture modification (see Table 3). Responses from all stakeholder groups indicated support for international standardization.

**Table 3:** Texture-modified food names and number of levels by world region

Region	Names (least to most modified)
Africa	Normal, Soft, chopped, puree/mashed, liquid/blender
Australia + New Zealand	Full/normal, soft, minced + moist, puree/smooth puree
Asia	Regular, soft, minced/shredded, congee/puree, liquidized/blenderized
Canada	Regular, soft, minced, puree
Europe	Normal, soft/tender/cut up, ground/puree, liquid
Ireland	Regular, soft, minced + moist, puree/smooth puree, liquidized
Middle East	Solid, soft, minced + mashed, other puree
South America	Solid, soft, mashed, thick puree, liquidized
United Kingdom	Normal, fork mashable/soft, pre-mashed/texture D, puree, thin puree
United States of America	Regular, advanced/stage 3, mechanical soft/chopped/stage 2, ground, puree/stage 1

*Note* 54 different labels were identified internationally for ≥5 food texture modification levels

The survey responses showed that some terms were not commonly used or familiar in all countries. For example, the terms “pudding,” “minced,” and “nectar” while understood by respondents from western cultures were not understood by respondents from Asia. Some currently used terms were considered to be problematic for certain populations. For example, it was noted that in the pediatric population and specifically children under 12 months of age, ‘honey from bees’ is contraindicated due to botulism risk. Thus, use of the term ‘honey thick’ was not felt to be an appropriate label for liquids served to pediatric populations. In addition, comments suggested that perceptions of honey differ considerably, as honey comes in crystalline, thick, and thin runny forms. Color coding was reported to be the most commonly used schema (53%) for differentiating different levels of thickened drinks or texture-modified foods; however, there was no congruency in colors chosen.

Of the respondents to the healthcare and food service professional stakeholder survey, 41 and 43%, respectively, reported that they test the consistency of foods and drinks to confirm suitability prior to serving. Consistency testing was more common among patients and caregivers, of whom, 57 and 60% reported testing foods and drinks, respectively. Visual inspection or observation was the most commonly used method of testing, regardless of stakeholder type. Patients, caregivers, and health professionals also reported using a spoon drop test or a utensil such as a fork for testing foods and liquids. Industry respondents, however, were most likely to assess liquids using a viscometer, Bostwick consistometer, or rheometer, in conjunction with visual inspection. For foods, industry respondents reported use of a texture analyzer, sieve, Bostwick consistometer, and visual inspection.

### Draft IDDSI Framework

**Fig. 4:**
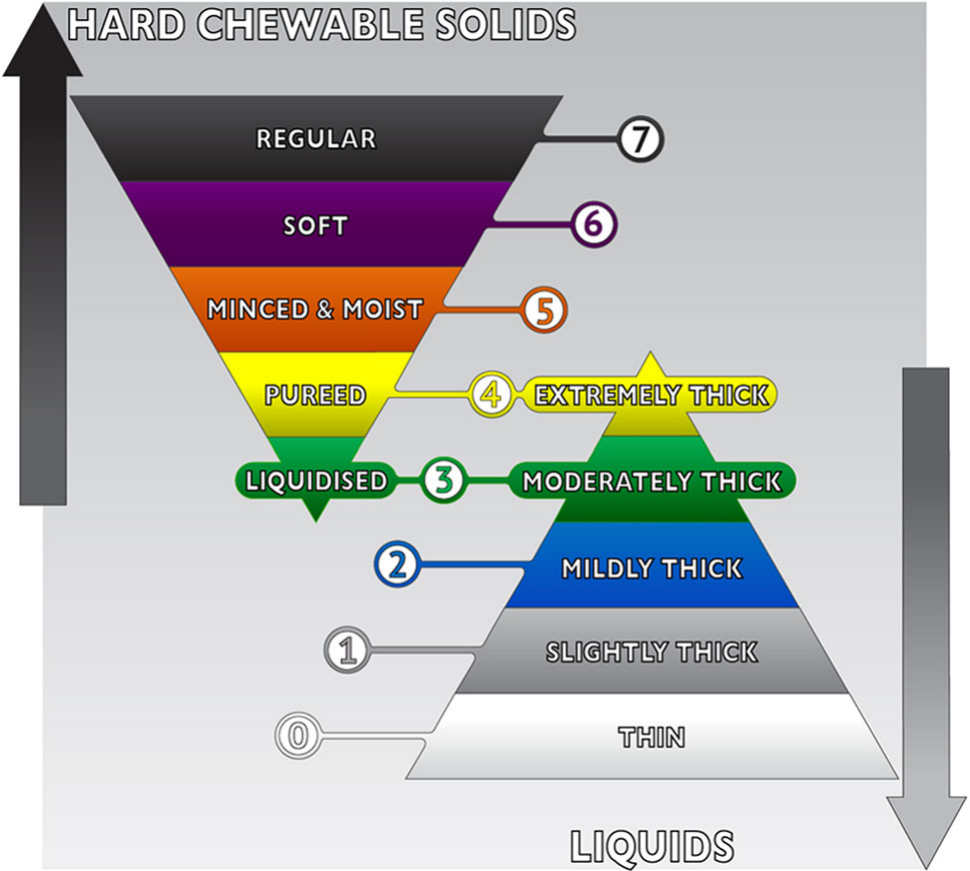
Draft pyramid image, highlighting the overlap zone

The draft framework resulting from the 2015 face-to-face meeting was represented as a continuum of 8 levels with foods and liquids displayed on a single scale using a twin-pyramid design showing foods in the top, inverted pyramid and liquids in the bottom, standing pyramid (see Fig. 4). The decision to use the pyramid image was partly influenced by the fact that a pyramid was already in use nationally in Japan for dysphagia diets. In addition to making decisions about the pyramid graphic, the number of levels, and the numbering scheme, the committee chose a draft color scheme with the aim of making each color as distinguishable as possible. It was decided that the color red should be avoided, given that red is frequently used as a color to denote alarm and danger in medical contexts and may also have other symbolism in some cultures.

A novel feature of the draft framework was the decision to recognize that certain food textures shared flow properties with thickened liquids creating an overlap zone in the middle of the framework. Using the same numbers to refer to both food and drink items at these levels, recognized the shared flow properties of these textures. Specifically, Level 3 was used both for Liquidized foods and Moderately Thick fluids, while Level 4 was used both for Pureed food and Extremely Thick fluids. All other levels had distinct flow or texture properties.

### Draft Definitions

The committee developed detailed definitions for each level of the draft Framework, based on (a) the measurement activities conducted at the 2015 face-to-face meeting; (b) drawing from descriptors in all available national standards documents; and (c) the literature describing properties that increase risk for choking [35–44]. The draft definitions included a warning after Level 6 to clarify that the physiological skills of being able to both bite and chew food were required to safely transition to Level 7 Regular foods. Physiological contraindications for advancing to Level 7 were listed, such as xerostomia, requirement for dentures, difficulty managing mixed textures, impulsive behavior, cognitive impairment, delayed oral skills (dentition, chewing development), and fatigue (impaired strength or stamina). A level that was tentatively titled ‘Level 7 Minus’ was included to capture food textures that are hard in their original state but break down quickly with moisture or temperature change and can then be manipulated with minimal chewing or just with tongue pressure.

### Draft Measurement Guidelines

#### Liquids

The 2015 face-to-face meeting included evaluation and discussion of the available testing methods for liquids: viscosity measurement was rejected due to being inaccessible in most situations and not necessarily capturing the important textural properties for swallowing (see “Discussion” section below). The draft IDDSI framework included a description of a gravity flow testing method for liquids using a syringe aiming to provide physiologically relevant flow conditions in a convenient, accessible, inexpensive test (see “Discussion”). An explanation of the gravity flow test was included in the survey to gauge acceptance of the method prior to final development. Stakeholder feedback indicated that the test was easy to understand and to implement. Detailed information about the gravity flow test is shown in “Final IDDSI Framework” section (see “Results” below).

### Foods

Formal assessment of food texture commonly requires complex and expensive machinery, such as Food Texture Analyzers. This type of assessment was rejected as a practical measurement option given the lack of access to food texture analyzers and expertise or interpretation. The draft framework did not include quantitative methods for testing food texture, although the committee agreed that a method to distinguish food into the various categories was highly desirable. Subsequent to stakeholder feedback on the draft framework the committee developed practical quantitative methods for testing food size and texture (see “Final IDDSI Framework” in “Results” for more details).

### Survey 2: International Feedback on Draft IDDSI Framework

**Fig. 5:**
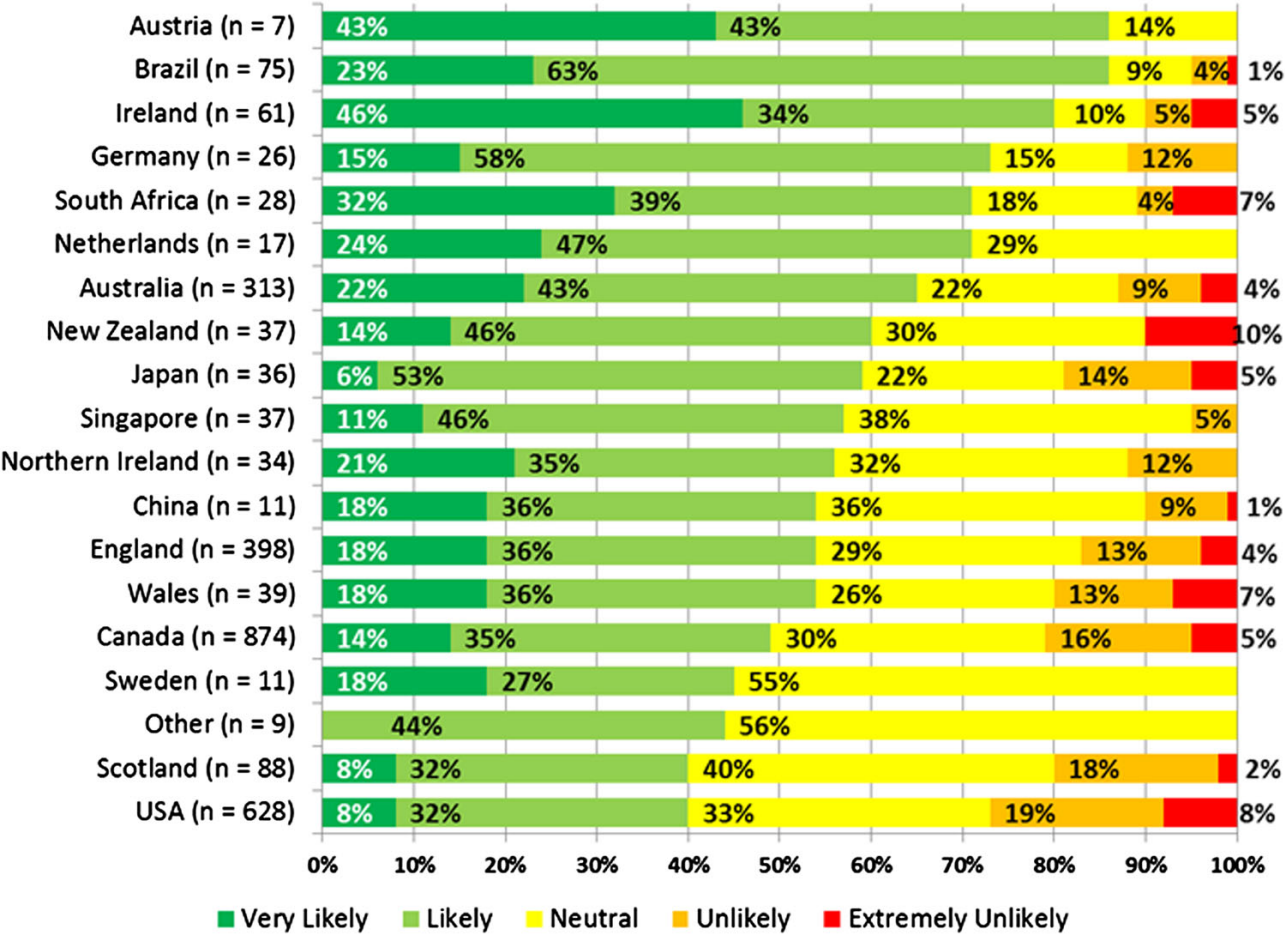
Stakeholder survey 2—International indications of likelihood of implementation. *Note* Caution should be used in interpreting small sample sizes from specific countries

The draft framework was submitted to international stakeholder consultation with a total of 3190 respondents residing in 57 different countries. The majority of respondents (87%) were health professionals working with dysphagia, although responses were also collected from caterers providing food to people with dysphagia, researchers/academics, industry that provides products to people with dysphagia, professional associations, government/regulatory bodies, caregivers to persons with dysphagia, and persons with dysphagia. Ninety percent of respondents came from English-speaking backgrounds and predominantly northern hemisphere countries. Fifty-three percent of respondents indicated that they or their organization were likely to implement the framework, with 28% neutral (see Fig. 5). Fewer than 19% of respondents indicated that implementation of the framework was unlikely.

Feedback regarding the colors representing the different food textures and thickened drink levels showed that they were considered easy to distinguish from each other and easy to implement. The number of levels was considered by more than two-thirds of respondents to be ‘about right’ and response to the twin-pyramid design was positive. Eighty percent of respondents rated the relevance and amount of information in the detailed definitions as ‘excellent’ or ‘good.’ Seventy-three percent of respondents indicated that the description of the syringe test was easy to understand. Clinicians who treated pediatric populations and people with developmental disability confirmed the need to have a category that included ‘meltable’ or ‘dissolvable’ solid foods. Forty percent of respondents agreed with the inclusion of Level 7 Minus with the same number neutral regarding its inclusion. The survey consultants (ASR) recommended that IDDSI review the framework based on the feedback received and make adjustments. This process of review and discussion occurred between June and November 2015.

### Final IDDSI Framework

**Fig. 6:**
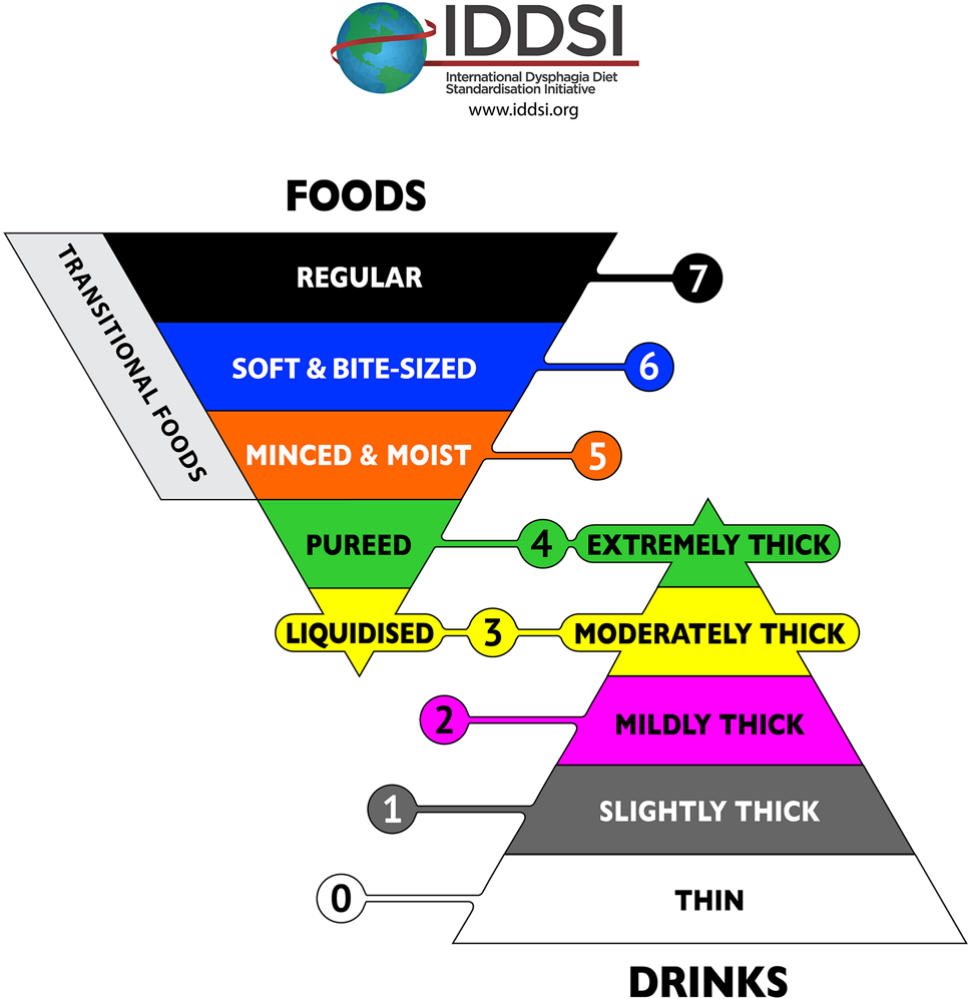
The final IDDSI framework graphic

The final framework is shown in Fig. 6. Notable changes from the draft to the final framework included delineation of the ‘transitional foods’ side-bar category to replace ‘Level 7 minus,’ changes to the color scheme and the inclusion of specific testing methods for foods.

The label ‘Level 7 Minus’ was deleted from the framework and replaced with the term ‘Transitional foods,’ running alongside Levels 5–7 on the inverted food pyramid. This location reflects the fact that transitional foods are regular foods (Level 7) with special textural properties such that with the application of moisture (e.g., saliva) or a change in temperature, they rapidly change their texture, crossing boundaries between levels. The colors were reviewed in detail and assessed for suitability for people with color blindness (e.g., protanopia, deuteranopia, tritanopia and monochromatism) to distinguish the framework colors. Based on the review, certain colors were changed to maximize the difference in color between neighboring levels. The final scheme has six colors plus black and white that are individually distinguishable across all the different types of color blindness tested and particularly for red blind and green blind, which is the most common variant [45]. Specifically, Level 0 is white; Level 1 is gray; Level 2 is pink; Level 3 is yellow; Level 4 is green; Level 5 is orange; Level 6 is blue; and Level 7 is black.

### Liquid Specifications and Measurement

**Fig. 7:**
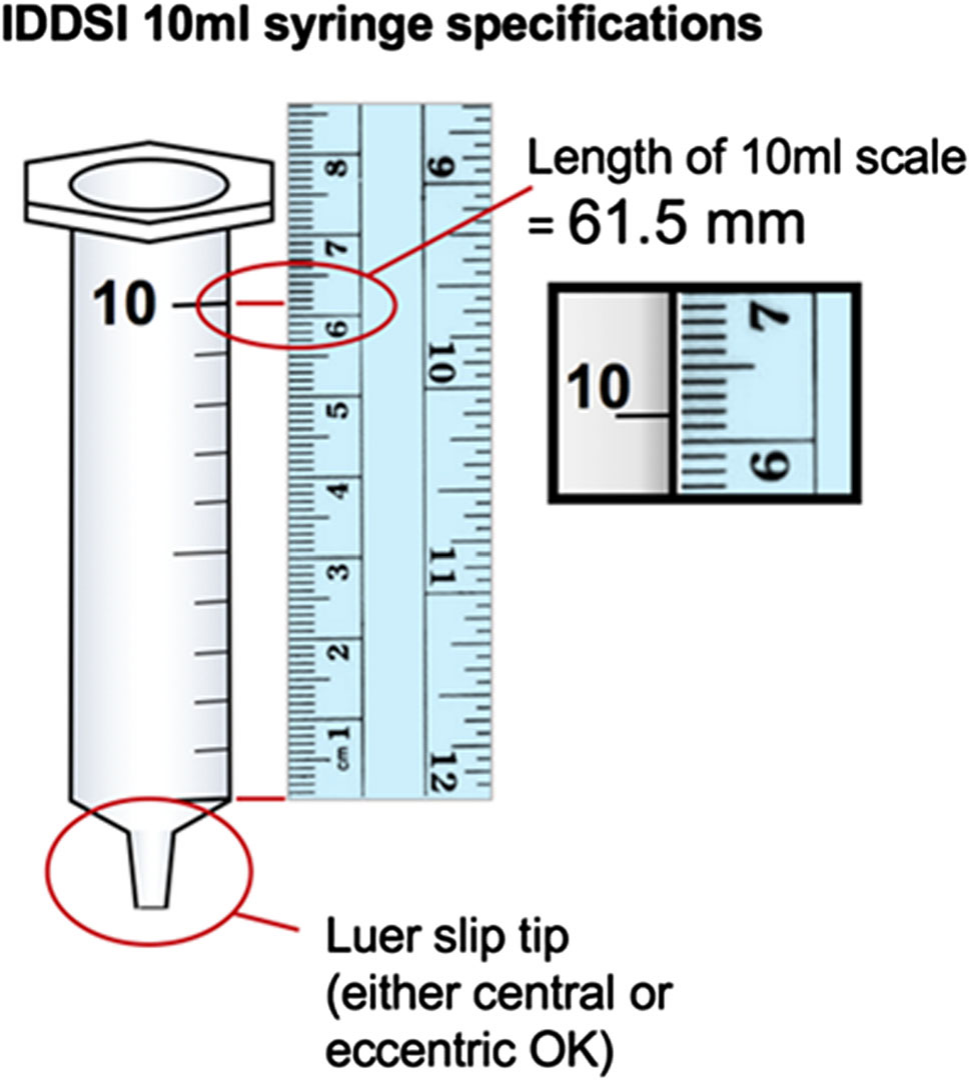
Example of a slip tip syringe that complies with IDDSI measurement requirements

The draft framework introduced the concept of the gravity flow test. The gravity flow test uses a 10-mL slip tip hypodermic syringe. Although 10-mL syringes were initially thought to be identical throughout the world based on reference to an ISO standard (ISO 7886-1) [46], it has subsequently been determined that the ISO document refers only to the nozzle of the syringe and that variability in barrel length and dimensions may exist between brands. As illustrated in Fig. 7, a syringe with a measured length of 61.5 mm from the zero line to the 10 mL line was used as the reference syringe (BD™ syringes were used for the development of the tests). To conduct the flow measurement, 10 mL of liquid is placed into an empty syringe and a stopper or finger is placed at the nozzle to impede flow until ready. When ready, the stopper or finger is removed from the syringe nozzle with flow allowed for 10 s. At 10 s, the nozzle is again blocked so that the volume of liquid remaining in the syringe can be recorded. The IDDSI Flow Test instructions and interpretations are included in the Appendix in supplementary material. During developmental testing by the committee, the IDDSI Flow Test was found to be suitable for thin liquids, naturally thick liquids and liquids thickened with a range of thickening agents (gums and starches) as well as items such as gravy, sauce, condiments, smooth soup, nutritional supplements, and liquid medication. Although the equipment is simple, the test has been found to categorize a wide range of liquids reliably in agreement with currently existing laboratory tests and expert judgment. It has been found to be sensitive enough to demonstrate small changes in thickness associated with change in serving temperature. The test requires that liquids are able to flow under their own weight, which corresponds to the threshold between level 3 and 4. While the test can be used to confirm whether a material is above the threshold for level 4 (no flow will occur), it is more convenient to simply use a spoon to determine whether the material is able to hold its shape or not. A number of countries use Fork Drip Tests to describe flow of thickened drinks or pureed food in their national terminologies [7, 17, 19]. Fork Drip Test criteria were developed for IDDSI Levels 3–5.

### Food Texture Specifications and Measurement

The systematic review demonstrated that the properties of hardness, cohesiveness, and slipperiness were important factors for consideration [27, 28]. In addition, as noted in the initial publication documenting the need for a new international framework, the size and shape of food samples have been identified as relevant factors for choking risk [26]. In view of this information, the IDDSI committee agreed that measurement of foods needed to capture *both* the mechanical properties (e.g., hardness, cohesiveness, adhesiveness, etc.) and the geometrical, size, or shape attributes of the food. Prior to release of the final framework, the committee worked to develop specifications based on the best available practical tools: the surveys had reported that utensils such as forks and spoons were commonly used for assessment of texture-modified food and thickened liquids. Assessments using chopsticks and finger tests have also been incorporated in recognition that these may be the most accessible methods in some countries.

### Food Particle Size

Assessment of foods requires a combination of evaluation for particle size and food hardness, cohesiveness, and adhesiveness. With regard to particle size, 2–4 mm represents the size of chewed particles that healthy adult individuals naturally masticate and reduce hard foods to for swallowing [47]. For Level 5 Minced & Moist, the recommended particle size for food served to adults is 4 mm. In recognition of the smaller anatomy and in lieu of pediatric research, for infants, the recommended particle size for Level 5—Minced & Moist food is 2 mm. The slots/gaps between the tines/prongs of a standard metal fork typically measure 4 mm, which provides a useful compliance measure for particle size of Minced & Moist foods served to adults.

For hard and soft solid foods served to adults, a maximum food sample size of ~1.5 × 1.5 cm is recommended, which is the approximate size of the adult human thumb nail [48] and the approximate width (from left to right] of the tip a standard metal fork. These dimensions represent the food texture industry standard ‘bite sample’ [47, 49], but most importantly are small enough to pass completely into the average adult trachea rather than obstruct it at the laryngeal inlet if accidentally inhaled [50, 51]. Tracheal size for adult males is 22 mm (range 15–27 mm) and for adult females is 17 mm (13–25 mm) [50]. Furthermore, food particle size of these dimensions has been identified as reducing asphyxiation risk [51].

Particle sizes for soft and hard food served to children younger than 5-year old are recommended to be no larger than 0.8 cm, which again relates to tracheal size and reduction of asphyxiation and choking risk [52]. Tracheal size of infants obviously changes as children grow. At age 20 months, the infant’s anteroposterior dimensions of the region just below the vocal cords, at the entrance to the trachea are approximately 3.8 mm × 6.5 mm. At 3 years 4 months (40 months), the dimensions are 7 mm x 3.9 mm and at 5 years of age the dimensions are approximately 8 mm × 4 mm [53]. It is for this reason that the Level 6—Soft & Bite-Sized specifies a particle size of 0.8 cm or less for children (i.e., 8 mm) and Level 5—Minced & Moist specifies a pediatric particle size of 0.2 cm (2 mm). Note also that food samples that are smaller than the maximum width of the child’s fifth fingernail (littlest finger) are unlikely to represent a choking risk, as this measurement is used to predict the internal diameter of an endotracheal tube in the pediatric population [54].

### Food Hardness, Cohesiveness, and Adhesiveness

Chewing results in the breaking down of food, determined by a number of factors including: toughness, moisture content of the food, ability to adsorb or absorb saliva, and the fibrous nature of the food [47, 55]. The level of moisture content in food has been particularly singled out as an important variable for determining food readiness for swallowing [55]. Salivation moistens the food bolus and assists with softening, disintegration, and dilution, thus reduced salivation will hinder even fully dentate individuals from adequately preparing a bolus for swallowing. During particle size reduction while chewing, the normal bolus is not ‘lump-free,’ however, it is moist and cohesive. For assessment of cohesiveness and adhesiveness a spoon tilt test is recommended. In each case the sample should (a) hold its shape on the spoon; and (b) fall easily from the spoon when tilted or turned sideways. There should be little residue left on the spoon. These characteristics provide a bolus that is moist and cohesive, but not sticky or adhesive.

**Fig. 8:**
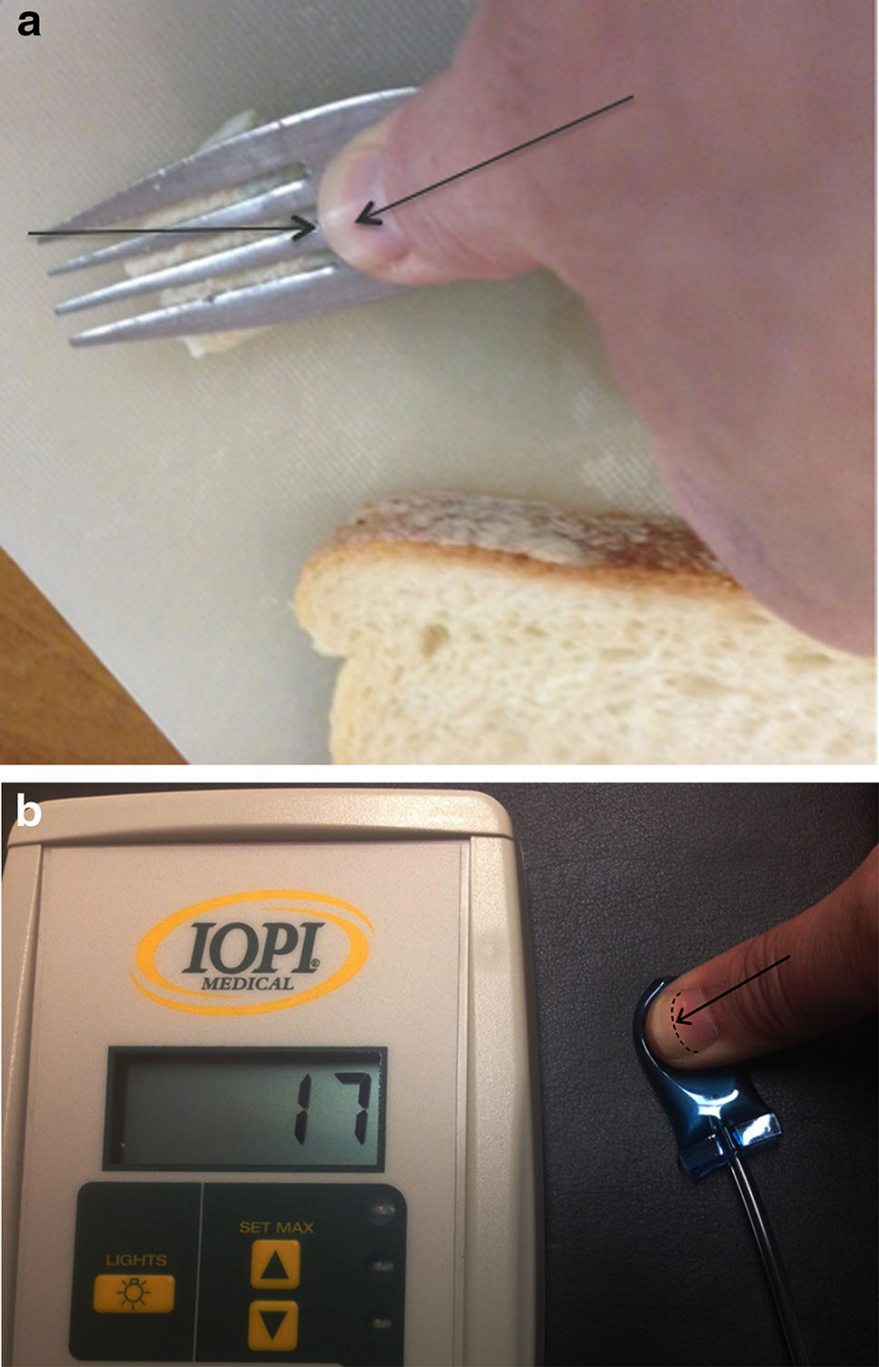
**a** Illustration of the thumb nail blanching to *white* (shown by *arrow*) during Fork Pressure Test. **b** Amount of pressure required (in kPa) to blanch the thumb nail to *white*. Image used with permission from IOPI Medical (www.iopimedical.com)

Quantification of food hardness is technically challenging because the mechanical structure of foods is generally complex. In industrial and scientific laboratories, a food texture analyzer is used to crush a sample of the food under controlled pressure and motion, but that requires motors and sensors. A practical test using a fork or spoon was previously recommended as part of the United Kingdom dysphagia diet standards [19] for assessing foods that would fall into IDDSI Levels 5–7 and transitional foods. The test involves applying a fork to the food sample to observe its behavior when pressure is applied, however, this varies with the level of force applied by the individual. In order to provide some standardization of the pressure applied, the IDDSI fork pressure test recommends that the fork be pressed onto the food sample by placing the thumb onto the bowl of the fork (just below the prongs), and pressing just hard enough to cause blanching of the thumbnail, Fig. 8a. Blanching occurs when the pressure overcomes mean arterial blood pressure and has been quantified at approximately 17 kPa, Fig. 8b. This pressure corresponds closely to a typical tongue pressure used during swallowing [56, 57]. In places where forks are not used, descriptions and testing methods have been developed for chopsticks and finger pressure testing.

To meet the requirements for Level 6—Soft & Bite-sized, a food sample should squash with the application of pressure and *not* return to its original shape when pressure is released. Transitional foods can also be identified using the Fork Pressure Test. For transitional foods, a sample 1.5 × 1.5 cm is placed in a container with 1 mL of water. Testing occurs after 1 min of food soaking has occurred. The sample qualifies as transitional food texture if the sample squashes and disintegrates and no longer resembles its original shape, or if it has melted significantly so that it no longer looks like its original shape.

Consistent with existing national terminologies and evidence from autopsy data, tables showing ‘texture requirements’ and ‘texture restrictions’ for each level were generated (see Appendix in supplementary material). Foods that have been identified in multiple autopsy reviews to increase choking risk were specifically addressed in a Frequently Asked Questions (FAQs) section (www.iddsi.org).

### Release of the Final IDDSI Framework

The final framework was released by staggered roll out. The framework design including the twin-inverted pyramid design was launched at the Japanese Society of Dysphagia Rehabilitation Conference September 2015. The detailed descriptors for drinks were released online and via poster at the European Society of Swallowing Disorders Conference in September 2015; and the detailed descriptors for foods were released online and at the Food for the Elderly Conference, Hangzhou, China, in November 2015. Further to the release of the framework and detailed descriptors, and following consultation with a representative from the Australian Government Open Access and Licensing Framework (AusGOAL) and Creative Commons Australia, the IDDSI Framework and detailed descriptors were licensed under the CreativeCommons Attribution Sharealike 4.0 Licence https://creativecommons.org/licenses/by-sa/4.0/legalcode to facilitate language translation.

To use the IDDSI framework and detailed descriptors, we request the following attribution:

© The International Dysphagia Diet Standardisation Initiative 2016 @http://iddsi.org/framework/. Attribution is NOT PERMITTED for derivative works incorporating any alterations to the IDDSI Framework that extend beyond language translation.

Supplementary Notice: Modification of the diagrams or descriptors within the IDDSI Framework is DISCOURAGED and NOT RECOMMENDED. Alterations to elements of the IDDSI framework may lead to confusion and errors in diet texture or drink selection for patients with dysphagia. Such errors have previously been associated with adverse events including choking and death.

## Discussion

The International Dysphagia Diet Standardisation Initiative utilized an evidence-based method of guideline development [24, 25] to produce new global standardized terminology and definitions to describe texture-modified foods and thickened liquids used for individuals with dysphagia of all ages, in all care settings and all cultures. The final framework was developed with reference to existing national terminologies, empirical data from multiple international stakeholder consultations of people from 57 countries, systematic review of the research literature and collaborative, feedback-driven refinement. Feedback about the framework was collated and analyzed by a research group independent to IDDSI further strengthening confidence in the refinement of the final framework. The final framework consists of eight levels (Levels 0–7) that are identified by numbers, text labels, and color codes. Text labels have been scrutinized for ease of translation and color codes have been developed to be sensitive to color blindness. Descriptors are supported by simple, accessible yet objective measurement methods that can be used by people with dysphagia and their caregivers, clinicians, food service professionals, researchers, and industry to confirm the level of attribution of a food or liquid. The IDDSI framework provides a solid platform for the development of future research in the dysphagia field.

The IDDSI framework provides categorization of liquid thickness levels applicable to neonates, infants, children, and adults with dysphagia. The IDDSI systematic review found evidence confirming that thickening liquids reduces the likelihood of aspiration, however, it was not able to pinpoint specific viscosities that represent minimally effective thickening to reduce aspiration. The review did, however, find evidence to suggest that some extremely thick liquids may promote the accumulation of pharyngeal residue [23, 27, 28, 58]. This finding has been further corroborated by Newman and colleagues [59], who conducted an independent systematic review of the literature on the efficacy of thickened liquids for the management of dysphagia. Recognition that some liquids may promote residue by being ‘too thick’ is an important development for the dysphagia field. Given the paucity of research regarding therapeutic thickness levels for thickened drinks, the IDDSI framework is based on an understanding that increasing thickness has a demonstrated therapeutic benefit for reducing the likelihood of penetration/aspiration. The number of levels of drink thickness included in the framework and recommended for best practice is based on the synthesis of international stakeholder consensus on current clinical practice. The systematic review points to an urgent need to conduct quality research to determine specific thickness levels that provide therapeutic benefit by reducing risk for penetration/aspiration and/or improving swallowing function. The IDDSI framework provides a reference point for this research and with future developments it is anticipated that the IDDSI levels will be refined to reflect new evidence regarding therapeutic thickness levels.

### International Food Textures for Dysphagia Management

The IDDSI framework provides categorization of food textures applicable to babies, infants, children, and adults with dysphagia. Children younger than three years of age, adults over 65 years of age, individuals with poor dentition, and those with neurological conditions are at high risk of death from asphyxiation on food [35, 60]. In healthy people, regardless of the initial state of the food, after oral processing and at the point of swallow initiation, the bolus is a cohesive mass. Texture modification mechanically alters the food prior to ingestion to the level that is required to promote safe swallowing of the bolus. The paucity of research into the therapeutic use of food texture modification for dysphagia management means that the recommendations in this document regarding food texture are based on an understanding that altering food texture modification has demonstrated a therapeutic benefit for reducing the risk of choking. Empirical evidence gathered from the current practice survey indicated that foods are commonly altered in both size (chopped, diced) and texture (soft, puree) to reduce choking risk. This practice is consistent with evidence in the literature specific to choking and asphyxiation risk, which reveals that food textures that pose the most risk are categorized according to texture, shape, and size. Specifically, foods that are described as hard or dry; chewy or sticky; crunch or crumbly; floppy; fibrous or ‘tough’; have husks; are stringy; round or long in dimension or consist of multiple or ‘dual’ textures are high choking risks [35–44]. Additional discussion regarding choking risk can be found in the IDDSI Foundation manuscript [26].

The IDDSI framework promotes strict adherence to both particle size and food texture requirements. For Level 6—Soft & Bite-Sized, international feedback has requested justification for the food particle size on this diet. To reduce choking risk, pre-cut food to 1.5 × 1.5 cm has been recommended. For easy reference, it has been determined that the width of a standard dinner fork (from left to right running perpendicular to the prongs) corresponds approximately to this 1.5 cm dimension. It is not possible to guarantee that a person with dysphagia will be able to cut food to this size, or that care staff or family will be available to pre-cut the food. Individuals with cognitive impairment are at increased risk of choking with poor ability to self-monitor food size and rate of ingestion [51, 61]. Some elderly people without a formal dysphagia diagnosis, but with fewer than 20 teeth, or with dentures may benefit from soft food for ease of mastication. These individuals do not strictly require the stringent particle size requirement described in Level 6—Soft & Bite-Sized, but perhaps they also do not strictly require a dysphagia diet. In these cases, it is suggested that facilities consider specifying soft options from a regular diet. This option should not be considered as part of the dysphagia diet. The testing methods outlined in the IDDSI Framework are generalizable to testing the softness of food texture in such circumstances. It should be noted that the loss of occlusal units affects bite force. Individuals with greater than 20 teeth (10 paired occlusal units) are reported to have normal bite force values of ~555 N. An exponential decline in bite force is observed with a reduction in the number of teeth, for example, 383 N for 10–19 teeth remaining; 180 N for 1–9 teeth remaining and 155 N for edentulous individuals [62]. Regardless of a formal dysphagia diagnosis, reduced bite force and poor masticatory efficiency increases choking risk [63].

### Accessible, Objective Testing Methods for Texture-Modified Liquids and Foods

To date, the measurement of fluid thickness in most national terminologies has been based on subjective methods such as flow through the prongs of a fork which has inherent variability [64]. Objective quantification was highly desirable but was challenging. The only national standard to recommend categorization of liquids according to quantified viscosity ranges is the National Dysphagia Diet (NDD), developed in the USA in 2002 [15]. However, the IDDSI committee considered that there were major practical and scientific limitations to viscosity measurement as follows:

- The lack of access to testing equipment and the expertise required to perform and interpret rheological testing.
- Viscosity is only one of a number of relevant parameters affecting liquid flow; others include density, yield stress, sample temperature, elasticity, and propulsion pressure [65–69].
- Drinks thickened with different thickening agents—or naturally thick—may have the same measurement of apparent viscosity at the specified test shear rate (e.g., NDD: 50 s^−1^) and yet may have very different flow characteristics in practice [27, 28, 70–73].
- The non-Newtonian nature of thickened drinks makes them impossible to characterize fully with only one viscosity measurement [70, 74, 75].
- In addition to variations in flow associated with drink characteristics, flow rates during swallowing are expected to differ depending on a person’s age and level of impairment of swallowing function [59].

For these reasons, a measurement of viscosity has not been included in the IDDSI descriptors. Instead, an objective and practical measurement has been selected by IDDSI to classify liquids based on their rate of flow under the action of gravity down a narrow tube with an orifice at the bottom. Such tests have a history in the dairy industry for studying oral perceptions of milk, cream, and yogurt (e.g., [76, 77]). The controlled dimensions selected are broadly representative of drinking through a straw or beaker and the regime of top-down flow through a narrow tube with exit through a small orifice has physiological parallels with bolus flow through the pharynx, with exit via the upper esophageal sphincter. This type of extensional flow (as opposed to shear) has been hypothesized to be more relevant to perception and to dysphagia [78–80]. Rather than specify a proprietary instrument, we have specified a common 10-ml syringe due to its increased availability and affordability globally. The syringe may be disposed after each use or washed and re-used; the 10 ml sample fluid would be discarded. The nature of the test means it is possible to measure drinks at the point of service. Although we would not expect this to be performed routinely, it does provide a standardized objective measure for training, auditing, and research. The ability to measure liquid thickness also provides opportunity to accurately audit thickness of sauces, condiments, soups, nutritional supplements, and liquid medications at the time of preparation and at the point of serving.

It is desirable to develop similar practical tools for quantifying food textures, however, food texture assessment provides more variables for assessment than drinks as both texture and size requirements are needed. Additionally, a degree of added pressure is required in order to deform the materials (which will not flow under gravity), and that is difficult to control in an inexpensive and globally standardized manner. The IDDSI Fork pressure test provides guidelines with greater quantification than would be achievable by text alone. The dimensions of common forks have been found to be fairly consistent internationally, which provides some ability to specify particle size, and a version of the test has been produced for chopsticks. However, it is acknowledged that further work in the area of food texture assessment for texture-modified foods is warranted.

### Limitations of the Current Study

The IDDSI process utilized online stakeholder surveys to gather empirical evidence. It is acknowledged that despite the range of stakeholder groups engaged that the sample size of the stakeholder groups was uneven. The largest group of respondents in both surveys was healthcare professionals. People with dysphagia and their caregivers made up the smallest stakeholder group. Often communication impairment accompanies dysphagia, and it is possible that communication impairment may have limited respondents’ ability to participate in the surveys. The surveys were heavily influenced by responses from English-speaking countries, with the top 10 countries of origin responses coming in order from Canada, USA, England, Australia, Scotland, Brazil, Ireland, New Zealand, Singapore, and Japan. The stakeholder groups consisted of motivated responders. It is noted that in the development of the Australian standardized terminology for texture-modified foods and fluids that differences were seen in the responses from motivated vs. targeted respondents [7]. Due to the scale of the IDDSI initiative, it was not possible to solicit responses from individuals or organizations that had not already volunteered to take part in the surveys. It could be argued that those motivated to respond will be more active in change management.

### Future Directions

The IDDSI framework has been well received by the international community. The IDDSI Board is in the process of developing materials and resources to assist interested parties to transition to the IDDSI framework (www.iddsi.org). The IDDSI web site aims to provide a large and up-to-date resource for the international community to share and discuss ideas and experiences relating to texture modification and adoption of the IDDSI framework. We hope this will include practical tips and guidance for local regions. The web site is a channel for stakeholders to feed back evidence of the success or limitations of the IDDSI framework across settings internationally.

The IDDSI framework is considered a living document such that it will be formally reviewed at specified intervals with new editions noted by updated version numbers and year of review. As research is conducted and technology continues to expand, it is anticipated that further refinements to the framework and detailed definitions will occur. The framework and detailed definitions will be formally reviewed in 2020 to ensure that the evidence base supporting the IDDSI framework remains current.

## Electronic supplementary material

Below is the link to the electronic supplementary material.

Supplementary material 1 (PDF 998 kb)
