# Supplementary material for: Development of International Terminology and Definitions for Texture-Modified Foods and Thickened Fluids Used in Dysphagia Management: The IDDSI Framework
Source: Dysphagia. 2016 Dec 2;32(2):293–314. doi: 10.1007/s00455-016-9758-y (PMC5380696; doi:10.1007/s00455-016-9758-y)
Supplement: Supplementary file 1 — Supplementary material 1 (PDF 998 kb) [file 455_2016_9758_MOESM1_ESM.pdf]

Appendix IDDSI Framework

IDDSI
International Dysphagia Diet Standardisation Initiative
www.iddsi.org

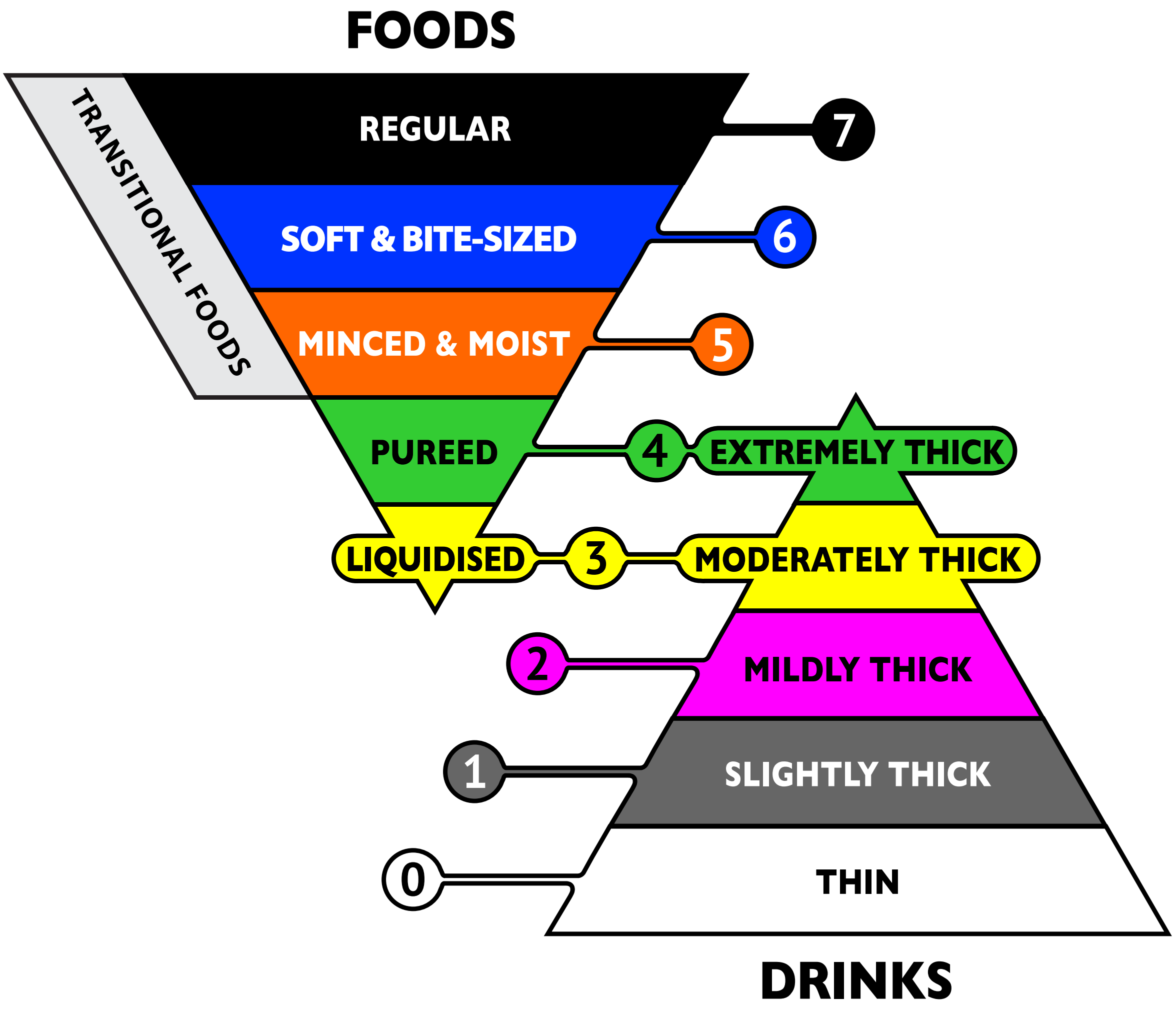

0 THIN

IDDSI
International Dysphagia Diet Standardisation Initiative
www.iddsi.org

Description/<br>Characteristics	• Flows like water<br>• Fast flow<br>• Can drink through any type of teat/nipple, cup or straw as appropriate for age and skills
Physiological rationale for this level of thickness	• Functional ability to safely manage liquids of all types
TESTING METHOD<br><br>IDDSI Flow Test*	• Test liquid flows through a 10 mL slip tip syringe completely within 10 seconds, leaving no residue (see IDDSI Flow Test instructions*)

1 SLIGHTLY THICK

IDDSI
International Dysphagia Diet Standardisation Initiative
www.iddsi.org

Description/<br>Characteristics	• Thicker than water<br>• Requires a little more effort to drink than thin liquids<br>• Flows through a straw, syringe, teat/nipple<br>• Similar to the thickness of commercially available 'Anti-regurgitation' (AR) infant formula
Physiological rationale for this level of thickness	• Predominantly used in the paediatric population as a thickened drink that reduces speed of flow yet is still able to flow through an infant teat/nipple. Consideration to flow through a teat/nipple should be determined on a case-by-case basis.
TESTING METHOD<br><br>IDDSI Flow Test*	• Test liquid flows through a 10 mL slip tip syringe leaving 1-4 mL in the syringe after 10 seconds (see IDDSI Flow Test instructions*)

2 MILDLY THICK

IDDSI
International Dysphagia Diet Standardisation Initiative
www.iddsi.org

Description/ Characteristics	• Flows off a spoon<br>• Sippable, pours quickly from a spoon, but slower than thin drinks<br>• Effort is required to drink this thickness through standard bore straw (standard bore straw = 0.209 inch or 5.3 mm diameter)
Physiological rationale for this level of thickness	• If thin drinks flow too fast to be controlled safely, these Mildly Thick liquids will flow at a slightly slower rate<br>• May be suitable if tongue control is slightly reduced.
TESTING METHOD<br>IDDSI Flow Test*	• Test liquid flows through a 10 mL slip tip syringe leaving 4 to 8 ml in the syringe after 10 seconds (see IDDSI Flow Test instructions*)

3 LIQUIDISED
3 MODERATELY THICK

IDDSI International Dysphagia Diet Standardisation Initiative www.iddsi.org

Description/characteristics<br><br>Texture restrictions shown in summary table	• Can be drunk from a cup<br>• Some effort is required to suck through a standard bore or wide bore straw (wide bore straw = 0.275 inch or 6.9 mm)<br>• Cannot be piped, layered or moulded on a plate<br>• Cannot be eaten with a fork because it drips slowly in dollops through the prongs<br>• Can be eaten with a spoon<br>• No oral processing or chewing required – can be<br>• swallowed directly<br>• Smooth texture with no 'bits' (lumps, fibers, bits of shell or skin, husk, particles of gristle or bone)
Physiological rationale for this level of thickness	• If tongue control is insufficient to manage Mildly Thick drinks (Level 2), this Liquidised/Moderately thick level may be suitable<br>• Allows more time for oral control<br>• Needs some tongue propulsion effort<br>• Pain on swallowing
TESTING METHOD<br><br>IDDSI Flow Test*	 <br>• Test liquid flows through a 10 ml slip tip syringe leaving > 8 ml in the syringe after 10 seconds (see Syringe Test Guide*)
Fork Drip Test	• Drips slowly in dollops through the prongs of a fork<br>• Tines/Prongs of a fork do <u>not</u> leave a clear pattern on the surface<br>• Spreads out if spilled onto a flat surface
Spoon Tilt Test	• Easily pours from spoon when tilted; does not stick to spoon
Chopstick Test	• Chopsticks are not suitable for this texture
Finger Test	• It is not possible to hold a sample of this food texture using fingers, however, this texture slides smoothly and easily between the thumb and fingers, leaving a coating

Food specific or<br>Other examples<br>(NB. this list is not exhaustive)	The following items may fit into this category:<br><br>• Infant “first foods” (runny rice cereal or runny pureed fruit)<br>• Sauces and gravies<br>• Fruit syrup

4 PUREED
4 EXTREMELY THICK

IDDSI
International Dysphagia Diet Standardisation Initiative
www.iddsi.org

Description/characteristics<br><br>Texture restrictions shown in summary table	• Usually eaten with a spoon (a fork is possible)<br>• Cannot be drunk from a cup<br>• Cannot be sucked through a straw<br>• Does not require chewing<br>• Can be piped, layered or molded<br>• Shows some very slow movement under gravity but cannot be poured<br>• Falls off spoon in a single spoonful when tilted and continues to hold shape on a plate<br>• No lumps<br>• Not sticky<br>• Liquid must not separate from solid
Physiological rationale for this level of thickness	• If tongue control is significantly reduced, this category may be easiest to manage<br>• Requires less propulsion effort than Minced & Minced (level 5), Soft & Bite-Sized (Level 6) and Regular (Level 7) but more than Liquidised/Moderately thick (Level 3)<br>• No biting or chewing is required<br>• Increased residue is a risk if too sticky<br>• Any food that requires chewing, controlled manipulation or bolus formation are not suitable<br>• Pain on chewing or swallowing<br>• Missing teeth, poorly fitting dentures
TESTING METHOD<br><br>IDDSI Flow test*	• No flow or drip through a slip tip syringe after 10 sec (refer to IDDSI Flow test instructions)*
Fork Pressure test	• The tines/prongs of a fork can make a clear pattern on the surface, and/or the food retains the indentation from the fork<br>• No lumps
Fork Drip test	• Sample sits in a mound/pile above the fork; a small amount may flow through and form a tail below the fork tines/prongs, but it does not flow or drip continuously through the prongs of a fork

Spoon Tilt test	• Cohesive enough to hold its shape on the spoon<br>• A full spoonful must plop off the spoon if the spoon is titled or turned sideways; a very gentle flick may be necessary to dislodge the sample from the spoon, but the sample should slide off easily with very little food left on the spoon; i.e. the sample should not be firm and sticky<br>• May spread out slightly or slump very slowly on a flat plate
Chopstick test	• Chopsticks are not suitable for this texture
Finger test	• It is just possible to hold a sample of this texture using fingers. The texture slides smoothly and easily between the fingers and leaves noticeable residue
Indicators that a sample is too thick	• Does not fall off the spoon when tilted<br>• Sticks to spoon
Food specific or Other examples	The following item may fit into this category:<br>• Purees suitable for infants (e.g. pureed meat, thick cereal)

5 MINCED & MOIST

IDDSI
International Dysphagia Diet Standardisation Initiative
www.iddsi.org

Description/characteristics<br><br>Texture restrictions shown in summary table	• Can be eaten with a fork or spoon<br>• Could be eaten with chopsticks in some cases, if the individual has very good hand control<br>• Can be scooped and shaped (e.g. into a ball shape) on a plate<br>• Soft and moist with no separate thin liquid<br>• Small lumps visible within the food<br>• *Paediatric, 2 mm lump size*<br>• *Adult, 4mm lump size*<br>• Lumps are easy to squash with tongue
Physiological rationale for this level of thickness	• Biting is not required<br>• Minimal chewing is required<br>• Tongue force alone can be used to break soft small particles in this texture<br>• Tongue force is required to move the bolus<br>• Pain or fatigue on chewing<br>• Missing teeth, poorly fitting dentures
TESTING METHOD<br><br>Fork Pressure test	 <br>• When pressed with a fork the particles should easily be separated between and come through the tines/prongs of a fork<br>• Can be easily mashed with little pressure from a fork [pressure should not make the thumb nail blanch to white]
Fork Drip test	• A scooped sample sits in a pile or can mound on the fork and does not easily or completely flow or fall through the tines/prongs of a fork
Spoon Tilt test	• Cohesive enough to hold its shape on the spoon<br>• A full spoonful must slide/pour off the spoon if the spoon is tilted or turned sideways or shaken lightly; the sample should slide off easily with very little food left on the spoon; i.e. the sample should not be sticky<br>• A scooped mound may spread or slump very slightly on a plate
Chopstick test	• Chopsticks can be used to scoop or hold this texture if the sample is moist and cohesive *and* the person has very good hand control to use chopsticks

Finger test	• It is possible to easily hold a sample of this texture using fingers; small soft, smooth, rounded particles can be easily squashed between fingers. The material will feel moist and leave fingers wet.
Food specific or<br>Other examples<br><br>Note:<br><br>*Paediatric, 2 mm lump size*<br>*Adult, 4mm lump size*	MEAT<br>• Finely minced or chopped, tender mince<br>• *Paediatric, 2mm lump size*<br>• *Adult, 4mm lump size*<br>• Serve in extremely thick, smooth, non-pouring sauce or gravy<br>• *If texture cannot be finely minced it should be pureed<br><br>FISH<br>• Finely mashed in extremely thick smooth, non-pouring sauce or gravy<br><br>FRUIT<br>• Serve mashed<br>• Drain excess juice<br><br>VEGETABLES<br>• Finely minced or chopped or mashed<br>• Drain any liquid<br><br>CEREAL<br>• Very thick and smooth with small soft lumps<br>• *Paediatric, 2mm lump size*<br>• *Adult, 4mm lump size*<br>• Texture fully softened<br>• Any milk/fluid must not separate away from cereal. Drain any excess fluid before serving<br><br>BREAD<br>• Pre-gelled 'soaked' breads that are very moist and gelled through the entire thickness<br>• No regular, dry bread<br><br>RICE<br>• Not sticky or glutinous (particularly short grain rice) and should not be particulate or separate into individual grains when cooked and served (particularly long grain rice)

6 SOFT & BITE-SIZED

Description/characteristics<br><br>Texture restrictions shown in summary table	• Can be eaten with a fork, spoon or chopsticks<br>• Can be mashed/broken down with pressure from fork, spoon or chopsticks<br>• A knife is not required to cut this food, but may be used to help loading a fork or spoon<br>• Chewing is required before swallowing<br>• Soft, tender and moist throughout but with no separate thin liquid<br>• 'Bite-sized' pieces as appropriate for size and oral processing skills<br>• *Paediatric, 8mm pieces*<br>• *Adults, 15 mm = 1.5 cm pieces*
Physiological rationale for this level of thickness	• Biting is not required<br>• Chewing is required<br>• Tongue force and control is required to move the food for chewing and to keep it within the mouth during chewing<br>• Tongue force is required to move the bolus for swallowing<br>• Pain or fatigue on chewing<br>• Missing teeth, poorly fitting dentures
TESTING METHOD<br><br>Fork Pressure test	 <br>• Pressure from a fork held on its side can be used to 'cut' or break this texture into smaller pieces<br>• When a sample the size of a thumb nail (~1.5x1.5 cm) is pressed with the base of a fork to a pressure where the thumb nail blanches to white, the sample squashes and changes shape, and does not return to its original shape when the fork is removed.
Spoon Pressure test	• Pressure from a spoon held on its side can be used to 'cut' or break this texture into smaller pieces.<br>• When a sample the size of a thumb nail (~1.5 cm x1.5 cm) is pressed with the bowl of a spoon, the sample squashes and changes shape, and does not return to its original shape when the spoon is removed.
Chopstick test	• Chopsticks can be used to break this texture into smaller pieces
Finger test	• Use a sample the size of the thumb nail (~1.5 cm x 1.5 cm). It is possible to squash a sample of this texture

	using finger pressure such that the thumb and index finger nails blanch to white. The sample will not return to its initial shape once pressure is released.
Food specific or<br>Other examples<br><br>Note:<br><br>*Paediatric, 8mm pieces*<br>*Adults, 15 mm = 1.5 cm pieces*	MEAT<br>• Cooked, tender meat no bigger than<br>• *Paediatric, 8mm pieces*<br>• *Adults, 15 mm = 1.5 x 1.5 cm pieces*<br>• If texture cannot be served soft and tender at 1.5 cm x 1.5 cm, serve minced and moist<br><br>FISH<br>• Soft enough cooked fish to break into small pieces with fork, spoon or chopsticks no larger than<br>• *Paediatric, 8mm pieces*<br>• *Adults, 15 mm = 1.5 cm pieces*<br>• No bones<br><br>CASSEROLE/STEW/CURRY<br>• Liquid portion must be thick<br>• Can contain meat, fish or vegetables if final cooked pieces are soft and tender and no larger than<br>• *Paediatric, 8mm pieces*<br>• *Adults, 15 mm = 1.5 cm pieces*<br>• No hard lumps<br><br>FRUIT<br>• Serve mashed<br>• Fibrous parts of fruit are not suitable<br>• Drain excess juice<br>• Assess individual ability to manage fruit with high water content (e.g. watermelon) where juice separates from solid in the mouth during chewing<br><br>VEGETABLES<br>• Steamed or boiled vegetables with final cooked size of<br>• *Paediatric, 8mm pieces*<br>• *Adults, 15 mm = 1.5 cm pieces*<br>• Stir fried vegetables are often too firm and not soft or tender<br><br>CEREAL<br>• Smooth with soft tender lumps no bigger than<br>• *Paediatric, 8mm pieces*<br>• *Adults, 15 mm = 1.5 cm pieces*<br>• Texture fully softened<br>• Any excess milk or fluid must drained

	BREAD<br>• Pre-gelled 'soaked' breads that are very moist and gelled through the entire thickness<br>• No regular dry bread unless assessed as suitable by dysphagia specialist, on an individual basis (if considered appropriate must also conform to paediatric 8mm, and adult 1.5 x1.5 cm size requirements)<br><br>RICE<br>• Not particulate/grainy, sticky or glutinous

7 REGULAR

Description/characteristics<br><br>There are NO texture restrictions at this level	• Normal, everyday foods of various textures that are developmentally and age appropriate<br>• Any method may be used to eat these foods<br>• Foods may be hard and crunchy or naturally soft<br>• Includes hard, tough, chewy, fibrous, stringy, dry, crispy, crunchy, or crumbly bits<br>• Includes food that contains pips, seeds, pith inside skin, husks or bones
Physiological rationale for this level of thickness	• Ability to bite hard or soft foods and chew them for long enough that they form a soft cohesive ball/bolus that is 'swallow ready'<br>• An ability to chew all food textures without tiring easily<br>• An ability to remove bone or gristle that cannot be swallowed safely from the mouth
TESTING METHOD	• N/A

TRANSITIONAL FOODS

IDDSI International Dysphagia Diet Standardisation Initiative
www.iddsi.org

Description/characteristics	• Food that starts as one texture (e.g. firm solid) and changes into another texture specifically when moisture (e.g. water or saliva) is applied, or when a change in temperature occurs (e.g. heating)
Physiological rationale for this level of thickness	• Biting not required<br>• Minimal chewing required<br>• Tongue can be used to break these foods once altered by temperature or with addition of moisture/saliva<br><br>➢ May be used for developmental teaching or rehabilitation of chewing skills (e.g. development of chewing in the paediatric population and developmental disability population; rehabilitation of chewing function post stroke)
TESTING METHOD<br><br>Fork pressure test	 <br>• After moisture or temperature has been applied, the sample can be easily deformed and does not recover its shape when the force is lifted.<br>• Use a sample the size of the thumb nail (~1.5 cm x 1.5 cm), place<br>1 ml of water on the sample and wait one minute. Apply fork pressure using the base of the fork until the thumbnail blanches to white. The sample is a transitional food texture if after removing the fork pressure:<br>• The sample has been squashed and disintegrated and no longer looks like its original state<br>• Or it has melted significantly and no longer looks like its original state (e.g. ice chips).
Spoon pressure test	• As above, using the bowl of the spoon in place of the fork
Chopstick test	• Use a sample the size of the thumb nail (~1.5 cm x 1.5 cm), place 1 ml of water on the sample and wait one minute. The sample should be easily broken apart using chopsticks with minimal pressure.

Finger test	• Use a sample the size of the thumb nail (~1.5 cm x 1.5 cm), place 1 ml of water on the sample and wait one minute. The sample will break apart completely by rubbing the sample between the thumb and index finger. The sample will not return to its initial shape
Food Specific or<br>Other examples<br><br><br><br><br><br><br><br><br><br><br><br><br>Specific examples used in paediatric or adult disability dysphagia management	This texture may include and is not limited to:<br><br>• Ice chips<br>• Ice cream/Sherbet if assessed as suitable by a Dysphagia specialist<br>• Japanese Dysphagia Training Jelly sliced 1 mm x 15 mm<br>• Wafers (also includes Religious Communion wafer)<br>• Waffle cones used to hold ice cream<br>• Some biscuits/ cookies/ crackers<br>• Potato crisps – only the mashed type (e.g. Pringles)<br>• Shortbread<br>• Prawn crisps<br><br><br>Commercially available foods that are transitional foods textures include but are not limited to:<br><br>• Veggie Stix™<br>• Cheeto Puffs™<br>• Rice Puffs™<br>• Baby Mum Mums™<br>• Gerber Graduate Puffs™

FOOD TEXTURE REQUIREMENTS

A green shaded check mark ☑ in the summary table below indicates a characteristic that is required and acceptable for foods in each level.

A red shaded ☒ in the summary table below indicates a food characteristic that is not acceptable for foods in each level.

DESCRIPTION/CHARACTERISTICS	3 Liquidised/ Moderately thick	4 Pureed/ Extremely thick	5 Minced & moist	6 Soft & bite-sized
No skin, no crust even after cooking, heating or standing	☑	☑	☑	☑
No separation of thin (watery) liquid	☑	☑	☑	☑
Will hold its shape on a plate, fork or spoon	☒	☑	☑	☑
Soft grainy texture quality	☒	☑	☑	☑
Visible lumps	☒	☒	☑	☑
Can contain soft, smooth, rounded, moist, small (2-4 mm) lumps if tender throughout	☒	☒	☑	☑
Can contain soft, moist large (8-15 mm) lumps if tender throughout	☒	☒	☒	☑

FOOD TEXTURE RESTRICTIONS

IDDSI
International Dysphagia Diet Standardisation Initiative
www.iddsi.org

A green shaded check mark ☑ in the summary table below indicates a characteristic that is acceptable and may be included for foods in each level.

A red shaded ☒ in the summary table below indicates a food characteristic that is not acceptable and must be avoided for foods in each level.

DESCRIPTION/CHARACTERISTICS	3 Liquidised/ Moderately thick	4 Pureed/ Extremely thick	5 Minced & moist	6 Soft & bite-sized	7 Regular
Mixed thin-thick textures (e.g. soup with pieces of food, cereal with milk; bubble tea)	☒	☒	☒	☒	☑
Hard or dry food (e.g. nuts, raw carrot, apple, crackling, hard crusty rolls)	☒	☒	☒	☒	☑
Fibrous or tough (e.g. steak, pineapple)	☒	☒	☒	☒	☑
Chewy (e.g. lollies/candies/sweets, cheese chunks, marshmallows, chewing gum, sticky mashed potato, dried fruits)	☒	☒	☒	☒	☑
Crispy (e.g. crackling, crisp bacon, cornflakes)	☒	☒	☒	☒	☑
Crunchy (e.g. raw carrot, raw apple, popcorn)	☒	☒	☒	☒	☑
Sharp or spiky (e.g. corn chips)	☒	☒	☒	☒	☑
Crumbly bits (e.g. crumbly dry cakes or biscuits)	☒	☒	☒	☑	☑
Pips, seeds, pith (e.g. apple seeds, orange pith)	☒	☒	☒	☒	☑
Skins or outer shells (e.g. peas, grapes)	☒	☒	☒	☒	☑
Husks (e.g. psyllium, bran)	☒	☒	☒	☒	☑
Skin (e.g. chicken skin, salmon skin)	☒	☒	☒	☒	☑
Bone or gristle (e.g. chicken bones, fish bones)	☒	☒	☒	☒	☑
Round, long shaped foods (e.g. sausage, grapes)	☒	☒	☒	☒	☑
Sticky or Gummy foods (e.g. nut butter, overcooked oatmeal, edible gelatin; Konjac containing jelly, sticky rice cakes)	☒	☒	☒	☒	☑
Stringy foods (e.g. beans, rhubarb)	☒	☒	☒	☒	☑

DESCRIPTION/CHARACTERISTICS	3 Liquidised/ Moderately thick	4 Pureed/ Extremely thick	5 Minced & moist	6 Soft & bite-sized	7 Regular
Hard pieces, skins or crusts formed during cooking or heating	☒	☒	☒	☒	☑
'Floppy' textures (e.g. lettuce, cucumber, baby spinach leaves)	☒	☒	☒	☒	☑
'Juicy' food where the juice separates from the solid in the mouth (e.g. watermelon)	☒	☒	☒	☒	☑

IDDSI Flow Test

www.iddsi.org

1

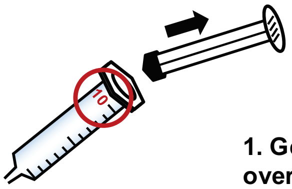

1. Get a stopwatch and some 10 mL syringes: see specification over the page. Remove the plunger from one syringe & discard.

2

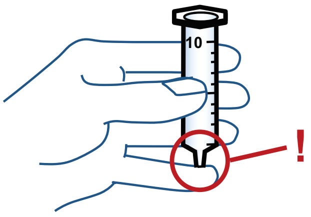

2. Cover the nozzle of the syringe with your finger, making a seal.

3

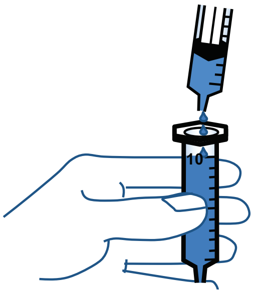

3. Fill the syringe up to the 10 mL line with fluid - it's recommended to use another syringe to do this.

4

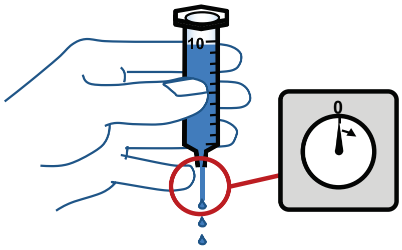

4. Remove your finger from the nozzle end at the same time as starting the stopwatch.

5

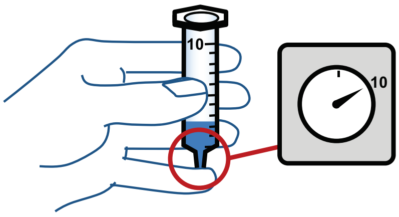

5. At 10 seconds, replace your finger over the nozzle, stopping the liquid flowing.

IDDSI Level classifications based on liquid remaining at 10 seconds:

Level 0: All liquid has flowed through syringe.

Level 1: There is between 1 mL and 4 mL remaining.

Level 2: There is between 4 mL and 8 mL remaining.

Level 3: There is more than 8 mL remaining, but some liquid still flows through.

Level 4: If no liquid flows, the category is Level 4 or above.

Level 4 can be easily identified without a syringe test: Material holds its own shape; small peaks remain on the surface. Too thick to be drunk from a cup or a straw, should be taken with a spoon. A full spoonful must drop off a spoon if turned sideways; a very gentle flick may be necessary but the material should not be firm, nor sticky.

© Ben Hanson 8/4/2016
